# Dermonecrosis caused by a spitting cobra snakebite results from toxin potentiation and is prevented by the repurposed drug varespladib

**DOI:** 10.1073/pnas.2315597121

**Published:** 2024-04-30

**Authors:** Keirah E. Bartlett, Steven R. Hall, Sean A. Rasmussen, Edouard Crittenden, Charlotte A. Dawson, Laura-Oana Albulescu, William Laprade, Robert A. Harrison, Anthony J. Saviola, Cassandra M. Modahl, Timothy P. Jenkins, Mark C. Wilkinson, José María Gutiérrez, Nicholas R. Casewell

**Affiliations:** ^a^Centre for Snakebite Research & Interventions, Department of Tropical Disease Biology, Liverpool School of Tropical Medicine, Liverpool L3 5QA, United Kingdom; ^b^Centre for Drugs & Diagnostics, Department of Tropical Disease Biology, Liverpool School of Tropical Medicine, Liverpool L3 5QA, United Kingdom; ^c^Department of Pathology and Laboratory Medicine, Queen Elizabeth II Health Sciences Centre and Dalhousie University, Halifax, NS B3H 1V8, Canada; ^d^Department of Applied Mathematics and Computer Science, Technical University of Denmark, Kongens Lyngby DK-2800, Denmark; ^e^Department of Biochemistry and Molecular Genetics, University of Colorado Denver, Aurora, CO 80045; ^f^Department of Biotechnology and Biomedicine, Technical University of Denmark, Kongens Lyngby DK-2800, Denmark; ^g^Instituto Clodomiro Picado, Facultad de Microbiología, Universidad de Costa Rica, San José 11501–2060, Costa Rica

**Keywords:** venoms, toxins, snakebite envenoming, neglected tropical diseases, drug repurposing

## Abstract

Snakebite envenoming is a neglected tropical disease that causes substantial mortality and morbidity globally. The venom of African spitting cobras often causes permanent injury via tissue-destructive dermonecrosis at the bite site, which is ineffectively treated by current antivenoms. To address this therapeutic gap, we identified the etiological venom toxins in *Naja nigricollis* venom responsible for causing local dermonecrosis. While cytotoxic three-finger toxins were primarily responsible for causing spitting cobra cytotoxicity in cultured keratinocytes, their potentiation by phospholipases A_2_ toxins was essential to cause dermonecrosis in vivo. This evidence of probable toxin synergism suggests that a single toxin-family inhibiting drug could prevent local envenoming. We show that local injection with the repurposed phospholipase A_2_-inhibiting drug varespladib significantly prevents local tissue damage caused by several spitting cobra venoms in murine models of envenoming. Our findings therefore provide a therapeutic strategy that may effectively prevent life-changing morbidity caused by snakebite in rural Africa.

## Significance

Spitting cobra venoms cause extensive local tissue damage surrounding the site of a snakebite. This damage cannot be effectively prevented with current antivenom treatments, and patients are often left with life-changing wounds. In this study, we used cellular and mouse experiments to determine which toxins in certain African spitting cobra venom are responsible for causing tissue damage, revealing that a combination of two different types of toxins is required to cause pathology in vivo. We then showed that the repurposed drug, varespladib, which targets one of these toxin types, effectively prevents skin and muscle damage in mouse models of envenoming. Collectively, these findings suggest that varespladib could be an effective type of therapy for preventing snakebite morbidity in Africa.

Snakebite is a neglected tropical disease (NTD) that primarily affects rural communities in sub-Saharan Africa, South/South-East Asia, and Latin America and causes an estimated 138,000 deaths per annum, with a further 400,000 people maimed annually (1). Although historically receiving little attention, in 2017 the World Health Organization (WHO) added snakebite to their list of priority NTDs and subsequently devised a roadmap aiming to halve the number of deaths and disabilities attributed to snakebite by 2030 (2).

Snakebite patients affected by local tissue damage often require surgical tissue debridement or amputation to prevent the onset of life-threatening gangrene. These severe sequelae greatly reduce the quality of life of many patients (3). Severe local pathology around the bite site results from cytotoxic, myotoxic, and/or hemorrhagic venom toxins, and is most often observed after viper envenoming (1, 4). While envenoming by most elapid snakes causes neurotoxic muscle paralysis and no local tissue damage, envenoming by several cobras (*Naja* spp.), most notably the African spitting cobras, causes little neurotoxicity but severe, rapidly developing swelling and tissue destruction that often leads to necrosis. These spitting cobra venoms also cause ophthalmia following defensive venom-spitting events (5–7). Spitting cobra bites are perhaps most frequent in sub-Sahel regions of Africa and include bites by *Naja pallida* in eastern Africa (8), *Naja mossambica* in southern Africa (9), and *Naja nigricollis*, which has a wide distribution throughout northern parts of sub-Saharan Africa (10). Collectively, envenomings by spitting cobras substantially contribute to the numerous cases of severe local envenoming that result in permanent, life-afflicting morbidity across the African continent (11).

The cobra venom toxins predominantly associated with causing dermonecrotic pathology are the cytotoxic three-finger toxins (3FTx), hereafter referred to as CTx, which make up 56 to 85% of the total toxin abundance in spitting cobra venoms (12). CTx are well known to disrupt cell membranes and/or induce pore formation (13–15), which leads to cell death through a series of intracellular events related to the loss of control of plasma membrane permeability and via direct interaction with organelles, such as lysosomes (13, 14). Although CTx are the most abundant toxin type found in many cobra venoms (12), it is usually only those of the spitting cobras that cause severe local tissue damage after envenoming (1), suggesting that additional toxins are likely contributing to the severity of local envenoming. The next most abundant toxin family in several cobra venoms are the phospholipases A_2_ (PLA_2_). While the PLA_2_ toxins found in elapid venoms are often neurotoxic (1), cytolytic PLA_2_ also exist which can cause tissue necrosis (16, 17). For example, the spitting cobra PLA_2_ nigexine is cytolytic toward multiple tumor cell lines and reduces cell viability and cell proliferation of epithelial human amnion cells (18). It has also been proposed that toxin combinations enhance venom cytotoxicity (19–21), with PLA_2_ toxins seemingly potentiating the effects of CTx (21). Understanding the relative contributions of different venom toxins to the severity of local envenoming is essential for the future design of targeted therapeutics to reduce the burden of snakebite morbidity—a key objective of our research.

Current treatment for snakebite envenoming relies on intravenous antivenom therapy, which consists of polyclonal antibodies generated via venom immunization of equines or ovines (1). While these therapeutics save countless lives, they are associated with several limitations that restrict their clinical utility, including low affordability to those in greatest need (1, 22), limited efficacy against a breadth of snake species due to venom toxin variation (22), and high incidences of severe adverse reactions in the case of some antivenoms (23, 24). The need to deliver antivenom intravenously by a medical professional in a clinical environment prolongs the time from bite to treatment by an average of 5 to 9 h due to poor hospital-accessibility in the remote, rural tropical regions where most snakebites occur (22, 25, 26). Furthermore, intravenous antivenom antibodies are too large (typically ~110 or ~150 kDa) to rapidly penetrate the envenomed peripheral tissue and neutralize the etiological cytotoxins—rendering antivenom treatment largely ineffective in reversing the swelling, blistering, and necrotic outcomes of local envenoming (1, 22, 23, 27, 28). Collectively, these limitations highlight why the development of effective therapeutics is one of the core goals of the WHO’s roadmap to reduce the impact of snakebite envenoming (2).

To address these therapeutic gaps, in this study we used a combined approach of in vitro cell cytotoxicity assays and in vivo murine models to quantify and identify the toxins responsible for venom-induced dermonecrosis caused by the most medically important African spitting cobras. Our findings demonstrate that CTx are largely responsible for cytotoxic effects observed in cellular assays using human epidermal keratinocytes, but that PLA_2_ toxins contribute extensively to in vivo envenoming pathology by working in conjunction with CTx to cause dermonecrosis. Using the PLA_2_-inhibiting repurposed drug varespladib (LY315920) (29–31), we then demonstrate significant reductions in venom-induced dermonecrotic pathology in vivo, suggesting that the local injection of PLA_2_-inhibitory molecules following envenoming is a viable therapeutic strategy to reduce lifelong morbidity caused by spitting cobra snakebites.

## Results

### Spitting Cobra Venoms Cause Heterogenous Dermonecrotic Lesions In Vivo.

To define the local envenoming pathology caused by medically important cobras, we intradermally challenged mice with venom from African spitting cobras. Mice were injected with two different doses of venom from East (Tanzania) and West (Nigeria) African forms of the black-necked spitting cobra (*N. nigricollis*), which collectively exhibit a broad distribution across sub-Saharan Africa and are known to induce severe local pathology in human victims (6, 10). After humanely killing the mice 72 h after venom challenge, the resulting dermonecrotic lesions were excised and analyzed macroscopically and microscopically.

**Fig. 1.**
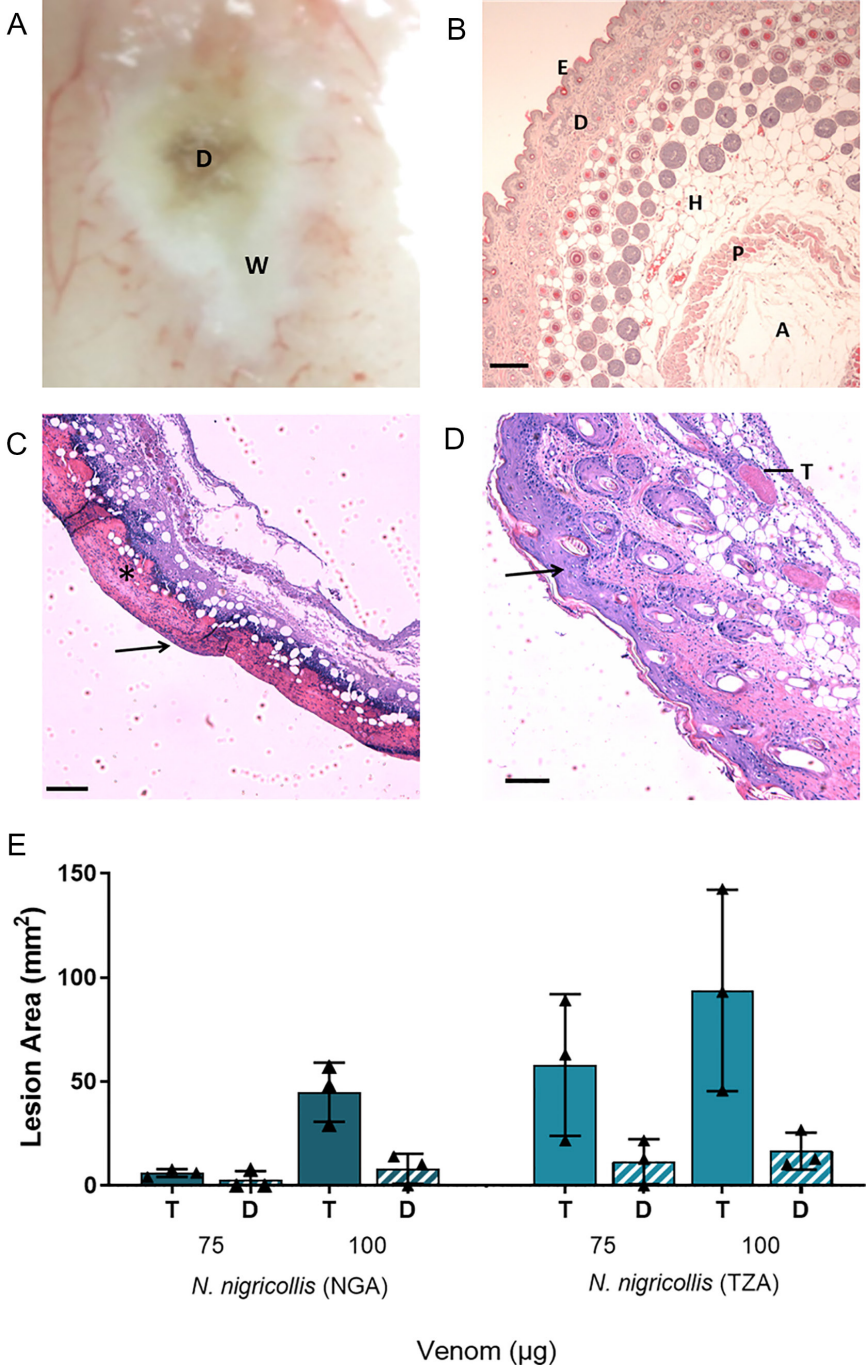
African spitting cobra venoms cause heterogenous dermonecrotic lesions in vivo. Groups of mice (n = 3) were injected intradermally with two doses of spitting cobra venom and after 72 h the resulting lesions were excised for macroscopic quantification of damaged areas and histological assessment. (*A*) Representative macroscopic image of a skin lesion induced by 100 µg of venom from West African (Nigeria) *N. nigricollis*, in which a dark central area (D) of necrosis is observed surrounded by a white area (W) of skin damage. (*B*–*D*) Representative light micrographs of sections of the skin of mice injected with PBS or West African *N. nigricollis* venom. (*B*) Skin injected with PBS showed a normal histological appearance including the epidermis (E), dermis (D), hypodermis (H), panniculus carnosus (P) and adventitia (A). (*C*) Light micrograph of a section of skin corresponding to a dark area of venom-induced damage. All skin layers were affected, with loss of epidermis (arrow) and skin appendages in the dermis. A proteinaceous hyaline material was observed (*). (*D*) Light micrograph of a section of the skin corresponding to a white area of damage from a mouse injected with venom. There was an increase in the thickness of epidermis (hyperplasia; arrow) and inflammatory infiltrate in the dermis. Thrombi (T) were observed in some blood vessels. (*E*) The area of dermonecrotic lesions caused by *N. nigricollis* (West African, Nigeria [NGA]; East African, Tanzania [TZA]) venoms at different doses. Bars show the mean area of the total lesions (T) in comparison to the dark central areas (D) of greatest intensity, and error bars represent the SD from the mean. Scale bar in (*B*–*D*) represent 100 µm.

Macroscopically, the lesions were generally heterogenous in appearance, presenting with a dark-colored necrotic center surrounded by a “white” area of tissue damage (Fig. 1*A*). To better define the lesion heterogeneity microscopically, we then performed histopathological analysis on hematoxylin and eosin (H&E) stained sections of the resulting lesions (Fig. 1 *B*–*D*). Sections from control mice receiving PBS only presented the typical histological pattern of normal skin, including epidermis, dermis (with skin appendages), hypodermis, panniculus carnosus, and adventitia (Fig. 1*B*). When areas of venom-induced skin damage were examined, there were clear histological differences between the macroscopically white and dark regions, with more pronounced damage observed in the latter. In samples collected from the dark lesions, there was extensive damage to all layers of the skin. The epidermis was lost and a hyaline proteinaceous material was observed, while the dermis and hypodermis were severely damaged with skin appendages absent. Moreover, there was widespread muscle necrosis in the panniculus carnosus (Fig. 1*C*). In contrast, the white lesions were characterized by hyperplasia of the epidermis and an inflammatory infiltrate in the dermis, together with thrombi in some blood vessels; though in general, the structure of the various layers of the skin was preserved and the skin appendages were present (Fig. 1*D*). Thus, these two different macroscopic patterns of skin lesions correspond to different histopathological scenarios. Last, the areas of the dark and total lesions were measured, revealing a general trend toward dose-dependent increases in lesion size, and that the area of the dark lesion never exceeded half of the total lesion area (Fig. 1*E*).

### Venom CTx Are Predominately Responsible for Cytotoxic Effects in Cell Culture.

**Fig. 2.**
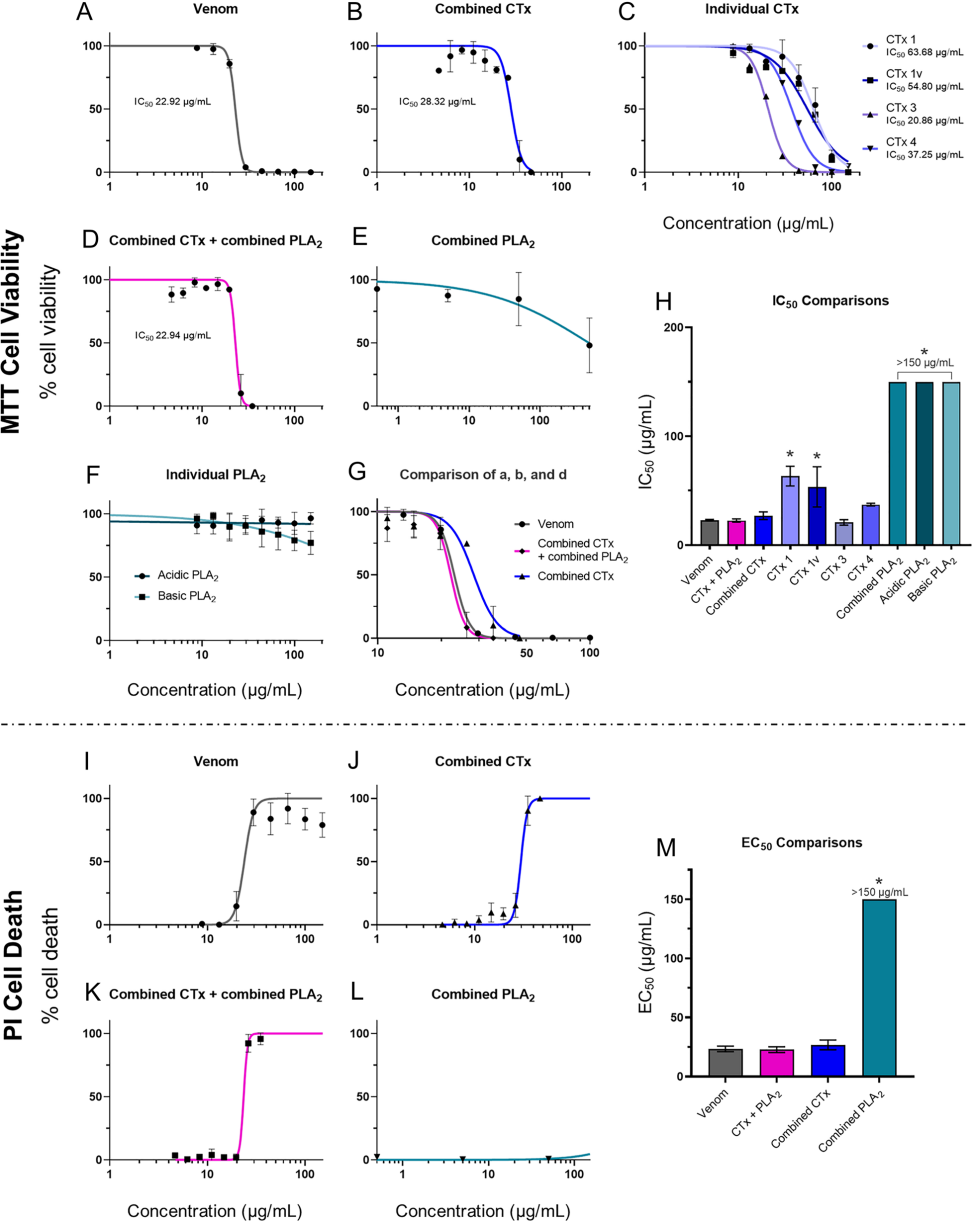
Crude venom and purified CTx inhibit cell viability, with CTx venom activity modestly potentiated by PLA_2_ toxins. Cell viability was measured in immortalized human keratinocytes (HaCaT cells) using MTT assays (*A*–*H*) and multiplexed with PI assays to measure cell death (*I*–*M*). HaCaT cells were treated for 24 h with serial dilutions of East African (Tanzania) *N. nigricollis* venom or its isolated toxins. MTT concentration–response curves are shown for (*A*) crude East African *N. nigricollis* venom, (*B*) combined purified CTx, (*C*) individual purified CTx, (*D*) combined CTx and combined PLA_2_ together, (*E*) combined purified PLA_2_, and (*F*) individual purified PLA_2_. (*G*) Direct comparison of the concentration–response curves caused by crude venom, combined CTx, and the combined CTx + combined PLA_2_ together. Note the different scale on the *x*-axis in comparison with panels *A*–*F*. (*H*) IC_50_ value summary of the various venom toxins displayed in (*A*–*F*) using MTT assays. PI concentration–response curves are shown for (*I*) crude *N. nigricollis* venom, (*J*) combined purified CTx, (*K*) combined CTx and combined PLA_2_ together, and (*L*) combined purified PLA_2_. (*M*) EC_50_ values of the various venom toxins displayed in (*I*–*L*) using PI assays. For panels *A*–*F*, the data shown represent mean % cell viability and corresponding SDs. For panels *I*–*L*, the data shown represent mean % cell death and corresponding SDs. All data displayed are from three independent experiments with each condition conducted in triplicate. Data were normalized to 0 to 100% between the lowest and highest read values for analysis, then plotted as concentration–response curves using GraphPad Prism 9. For panels *H* and *M*, statistically significant differences determined by one-way ANOVAs with Dunnett’s multiple comparisons post hoc tests are denoted by asterisks: *P < 0.05.

To identify which toxins in spitting cobra venom are responsible for causing the dermonecrotic effects observed in vivo, we first identified and quantified the cytotoxic potency of venom constituents using cell cytotoxicity methods in immortalized human epidermal keratinocytes (HaCaT cell line). This work was performed using East African (Tanzania) *N. nigricollis* venom, as this venom displayed more severe lesions than the West African (Nigeria) form in our earlier experiment, however, as *Naja* spitting cobra species across Africa share similar venomic profiles (21, 32), our results are likely relevant to the venoms of other related species as well. The venom was separated into its distinct constituents via gel filtration and cation exchange chromatography, followed by further purification using hydrophobic interaction or hydroxyapatite chromatography (*SI Appendix*, Figs. S1–S9). The identity of the isolated toxins was confirmed by mass spectrometric analysis (*SI Appendix*, Table S1). The HaCaT cells were then exposed to either crude venom, four purified CTx (CTx1 [UniProt: P01468], CTx1v [UniProt: P01468], CTx3 [UniProt: P0DSN1], and CTx4 [UniProt: P01452]), or two purified PLA_2_ (basic [UniProt: P00605] and acidic [UniProt: P00602]), and combinations consisting of all CTx (at a ratio reflective of relative abundance in the venom; ~3:1:1:1), all PLA_2_ (1:1), and all CTx and PLA_2_ together in a 2:1 ratio [reflective of that found in the crude venom (32)]. Following venom exposure, we performed thiazole blue tetrazolium (MTT) assays to assess cell viability via measures of metabolic activity (33, 34) multiplexed with propidium iodide (PI) assays as an indicator of cell death associated with plasma membrane disruption (35) (Fig. 2).

MTT measurements of cell viability, taken after 24 h of treatment, showed that crude venom potently reduced cell viability (IC_50_ 22.9 μg/mL ± 0.7; Fig. 2*A*). Similarly, all four of the purified venom CTx reduced cell viability, with CTx3 being the most potent (IC_50_ 20.8 μg/mL ± 2.5), followed by CTx4 (IC_50_ 37.2 μg/mL ± 1.2), and then CTx1 and CTx1v, which showed similar potencies (IC_50_ 63.4 μg/mL ± 9.0 and 53.5 μg/mL ± 18.4, respectively) and were significantly less potent than CTx3 (*P* = 0.004 and *P* = 0.020, respectively; Fig. 2 *C* and *H*). While the basic PLA_2_ visibly showed some cell viability inhibitory effects at the highest concentrations tested (≥100 μg/mL), neither of the two purified PLA_2_ alone (Fig. 2*F*) or combined in a 1:1 ratio (Fig. 2*E*) were sufficiently toxic to the cells to allow for the calculation of IC_50_ values. The combination of the four purified CTx gave a complete concentration–response curve with a resulting IC_50_ value approaching those obtained with crude venom (27.0 μg/mL ± 3.6 vs. 22.9 μg/mL ± 0.7, respectively; Fig. 2*B*), though remained slightly right-shifted in comparison (Fig. 2*G*). When the CTx and PLA_2_ combinations were pooled together in a 2:1 ratio, reflective of their toxin abundance in crude East African *N. nigricollis* venom (32), the resulting concentration–response curve became indiscernible from that of the crude venom and resulted in a near-identical IC_50_ value (22.6 μg/mL ± 1.5 vs. 22.9 μg/mL ± 0.7, respectively) (Fig. 2*H*).

PI measurements of cell death taken 24 h posttreatment with the various toxin combinations and East African *N. nigricollis* venom showed similar patterns (Fig. 2 *I*–*M*). The crude venom displayed potent cytotoxic effects resulting in EC_50_ values of 23.4 μg/mL ± 2.4, while the PLA_2_ combination did not cause sufficient cell death at the highest concentrations tested to calculate an EC_50_ value (Fig. 2 *I*, *L*, and *M*). The CTx combination resulted in an EC_50_ value close to that of the crude venom (EC_50_ of 26.8 μg/mL ± 4.1 vs. 23.4 μg/mL ± 2.4, respectively), while the 2:1 ratio of CTx:PLA_2_ combinations together modestly decreased the EC_50_ value (22.8 μg/mL ± 2.4), but to levels highly comparable to those obtained with crude venom (Fig. 2 *J*, *K*, and *M*).

### The Combination of Purified CTx and PLA_2_ Induces Venom-Induced Dermonecrosis In Vivo.

To understand whether CTx are also predominately responsible for dermonecrotic venom activity in vivo, we performed comparative experiments in our murine preclinical model of envenoming (36). The minimum necrotic dose of East African *N. nigricollis* venom, i.e., the dose that induces a lesion in the skin of 5 mm diameter 72 h after injection (36), was determined to be 63 µg/mouse, and doses of purified CTx, PLA_2_, and CTx + PLA_2_ that reflect their relative mass contribution to the total crude venom protein were determined [CTx and PLA_2_ comprise approximately 60% and 26% by weight of *N. nigricollis* venom, respectively (16, 32, 37–39)]. Thus, groups of mice received intradermal injections of either 63 μg of crude venom, 37.8 μg of the purified CTx combination, 16.4 μg of the purified PLA_2_ combination, or 37.8 μg plus 16.4 μg of the purified CTx and the PLA_2_ combinations, respectively, combined. After 72 h, animals were humanely killed, dermonecrotic lesions excised, measured, photographed, and processed for histopathological analysis (images of the resulting dermonecrotic lesions are presented in *SI Appendix*, Table S2).

**Fig. 3.**
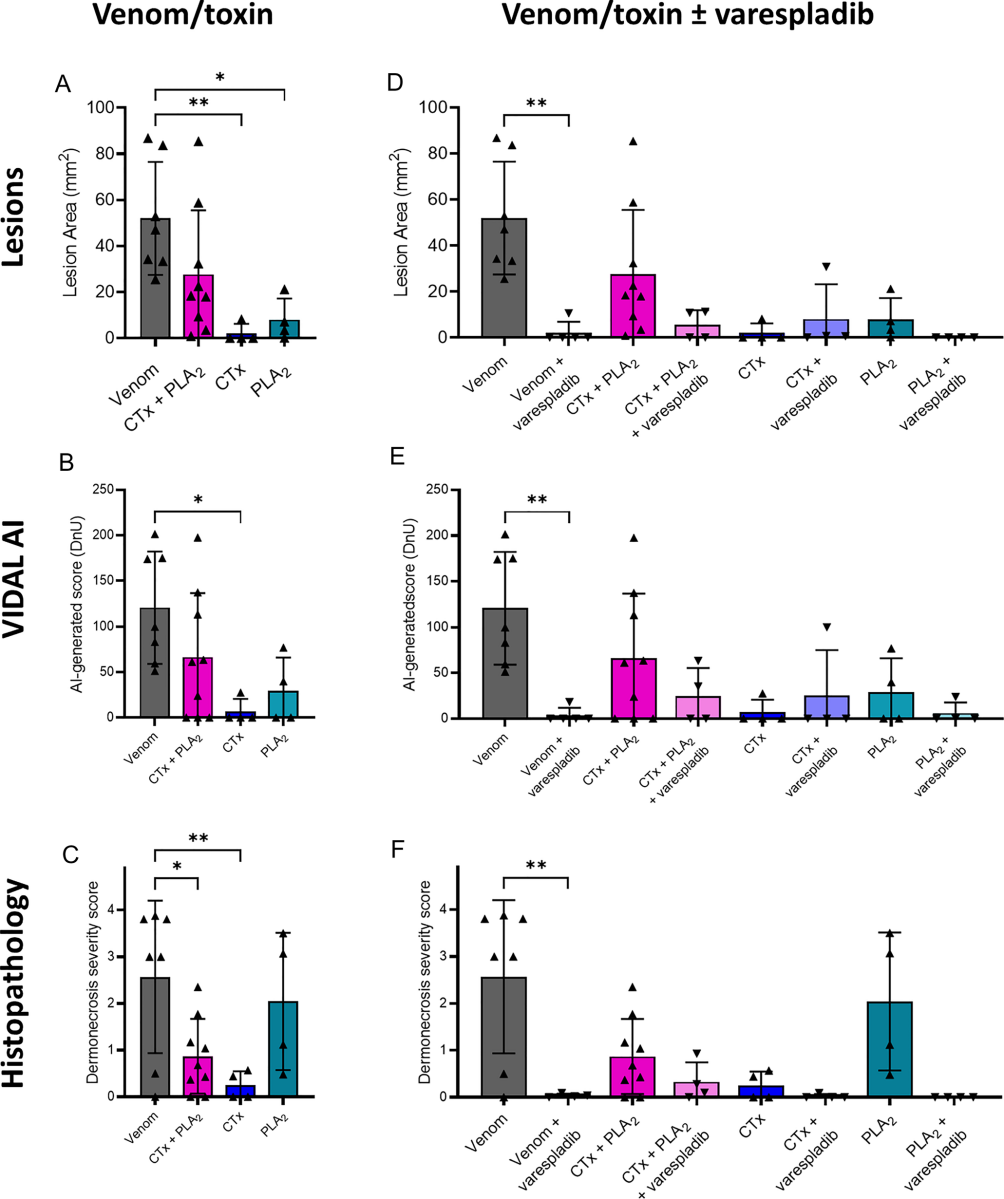
Spitting cobra venom causes dermonecrosis in vivo via CTx and PLA_2_ toxin potentiation, and inhibition of PLA_2_ toxins with varespladib reduces dermonecrosis severity. Groups of mice (n ≥ 4) were intradermally injected with either East African (Tanzania) *N. nigricollis* venom or purified venom constituents (CTx, PLA_2_, or a combination of CTx + PLA_2_) at doses reflecting their relative abundance in crude venom, with or without the PLA_2_-inhibiting small molecule drug varespladib (19 μg). At 72 h postinjection, lesions were excised and examined macroscopically and histopathologically. (*A*) A combination of venom CTx and PLA_2_ was required to recapitulate the dermonecrotic activity of crude *N. nigricollis* venom, as CTx and PLA_2_ toxins alone did not cause extensive dermonecrosis, as quantified via (*A*) caliper-measurements of lesion height and width, and (*B*) the lesion severity measuring AI tool, VIDAL. (*C*) Histopathological analysis of excised lesions showed similar results, except for a more severe dermonecrotic effect of the PLA_2_ alone. Preincubation with the PLA_2_ inhibitor varespladib reduced dermonecrotic lesion severity caused by East African *N. nigricollis* venom with similar trends for CTx + PLA_2_ and PLA_2_, as quantified with (*D*) calipers, (*E*) VIDAL, and (*F*) histopathological analysis. For damage scores of individual skin layers, see *SI Appendix*, Fig. S10. For panels *A* and *D*, the data shown represent mean lesion areas and corresponding SDs. Panels *B* and *E* show the mean lesion severity, as determined by VIDAL. Panels *C* and *F* show mean dermonecrosis severity scores and corresponding SDs calculated from those of the individual skin layers (*SI Appendix*, Fig. S10). Statistically significant differences were determined by one-way ANOVAs followed by Tukey's multiple comparisons post hoc tests and are denoted by asterisks: *P < 0.05, **P < 0.01. Error bars represent SDs.

Mean lesion areas resulting from venom injection were large and varied extensively among the experimental animals (52.0 mm^2^ ± 24.6) (Fig. 3*A*). Mice receiving the CTx and PLA_2_ combinations together (CTx + PLA_2_) developed lesions that did not differ significantly in size from those induced by the whole venom (27.7 mm^2^ ± 27.8; *P* > 0.05), suggesting that these two groups of toxins collectively are responsible for recapitulating much of the effects of crude venom. In contrast with our cytotoxicity data, however, the CTx combination alone resulted in negligible dermonecrosis in vivo, with only one of the four experimental animals displaying a visible lesion, and the mean lesion size (2.1 mm^2^ ± 4.1) being significantly lower than that caused by the crude venom (*P* = 0.027). Again, in contrast to the cell data, the PLA_2_ toxin combination resulted in three of the four mice developing visible lesions (8.0 mm^2^ ± 9.3), though the mean lesion size remained more than threefold lower than that observed with the crude venom and the CTx and PLA_2_ combination together. The overall severity of the lesions was also assessed using our recently developed, AI-based dermonecrosis quantification tool, VIDAL, which standardizes and quantifies lesion size and intensity to calculate an overall dermonecrosis score in Dermonecrosis Units (DnU), and provides a user-bias free method for quantifying lesions (*SI Appendix*, Fig. S11) (40). Quantification by VIDAL largely recapitulated the results above (Fig. 3*B*), confirming that lesion severity caused by crude venom (120.6 dermonecrotic units [DnU] ± 61.6) was not significantly different from that of the CTx + PLA_2_ combination (66.3 DnU ± 70.2), and that lesions caused by the CTx combination resulted in significantly less dermonecrosis (6.8 DnU ± 13.7, *P* = 0.025) than the crude venom. Last, total dermonecrosis scores (41) were calculated after histopathological assessment of H&E-stained lesion cross-sections for each animal (Fig. 3*C*). These data revealed that mice injected with crude venom showed extensive damage in all layers of the skin (dermonecrosis severity score of 2.6 ± 1.6). The PLA_2_ treated mice exhibited the next highest dermonecrosis severity scores (2.0 ± 1.5), followed by those receiving the CTx + PLA_2_ combination (0.9 ± 0.8) and then the CTx combination only (0.3 ± 0.3). These latter two groups exhibited dermonecrosis severity scores that were significantly lower than that of the crude venom (*P* = 0.0218 and *P* = 0.0091, respectively). Scoring for individual skin layers can be seen in *SI Appendix*, Fig. S10.

### The PLA_2_ Inhibitor Varespladib Protects against Venom-Induced Dermonecrosis.

Since our data demonstrated that venom-induced dermonecrosis relies on the combined effect of CTx and PLA_2_ venom toxins working together, we hypothesized that inhibiting just one of these toxin classes could significantly reduce the severity of venom-induced dermonecrosis in vivo. To that end, we repeated the experiments described above in the presence of the PLA_2_ inhibitor varespladib. Varespladib was originally designed for use in the treatment of cardiovascular diseases (42–44), but has recently entered phase II clinical trials for snakebite envenoming (45) following demonstration of its ability to prevent PLA_2_ toxin-driven systemic envenoming pathologies in animal models (29, 30, 46, 47).

We preincubated 19 μg of varespladib (41) with the same venom or purified toxin challenge doses before intradermally coinjecting mice and excising and analyzing lesions 72 h later, as described above. The coinjection of varespladib with crude venom caused statistically significant reductions in lesion sizes from 52.0 mm^2^ (±24.6) to 2.6 mm^2^ (±5.3) (*P* = 0.001; Fig. 3*D*). Coinjection of varespladib with the CTx + PLA_2_ combination also caused a substantial reduction in mean lesion size from 27.7 mm^2^ (±27.8) to 5.5 mm^2^ (±6.4), although this reduction was not statistically significant. Unsurprisingly, varespladib did not affect the minor lesion formation observed in the group dosed with the CTx combination, though when varespladib was dosed alongside the purified PLA_2_, the resulting lesion sizes decreased from a mean of 8.0 mm^2^ (±9.3) to no lesions being formed in any of the four experimental animals. These results were confirmed with the AI-generated dermonecrosis severity scores (Fig. 3*E*), from which the crude venom-induced lesions of 120.6 DnU ± 61.6 decreased significantly to 3.6 DnU ± 8.1 (*P =* 0.0054) when coincubated with varespladib. Similar effects of varespladib were seen when varespladib was coincubated with the CTx + PLA_2_ combination (decreasing mean lesion score from 66.3 DnU ± 70.2 to 24.7 DnU ± 30.7) and PLA_2_ (decrease from 29.2 DnU ± 36.9 to 5.9 DnU ± 11.8), albeit these were not statistically significant. Decreases in lesion severity were not observed when varespladib was cotreated with CTx. Histopathological analyses of skin lesion cross-sections also correlated with the macroscopic assessment of dermonecrosis. Mice receiving venom preincubated with varespladib showed significantly less microscopic damage than those that received venom alone (dermonecrosis severity scores: venom, 2.6 ± 1.6; venom and varespladib, 0.0 ± 0.0; *P* = 0.0035) (Fig. 3*F*). Reductions in microscopic damage by varespladib were also observed in animals receiving either the PLA_2_ + CTx combination or PLA_2_ dose, although these were not statistically significant (dermonecrosis severity scores: PLA_2_ + CTx, 0.9 ± 0.8 vs. PLA_2_ + CTx and varespladib, 0.3 ± 0.4; PLA_2_, 2.0 ± 1.5 vs. PLA_2_ and varespladib, 0.0 ± 0.0). Full details of the dermonecrosis severity scores obtained across each individual skin layer are presented in *SI Appendix*, Fig. S10.

**Fig. 4.**
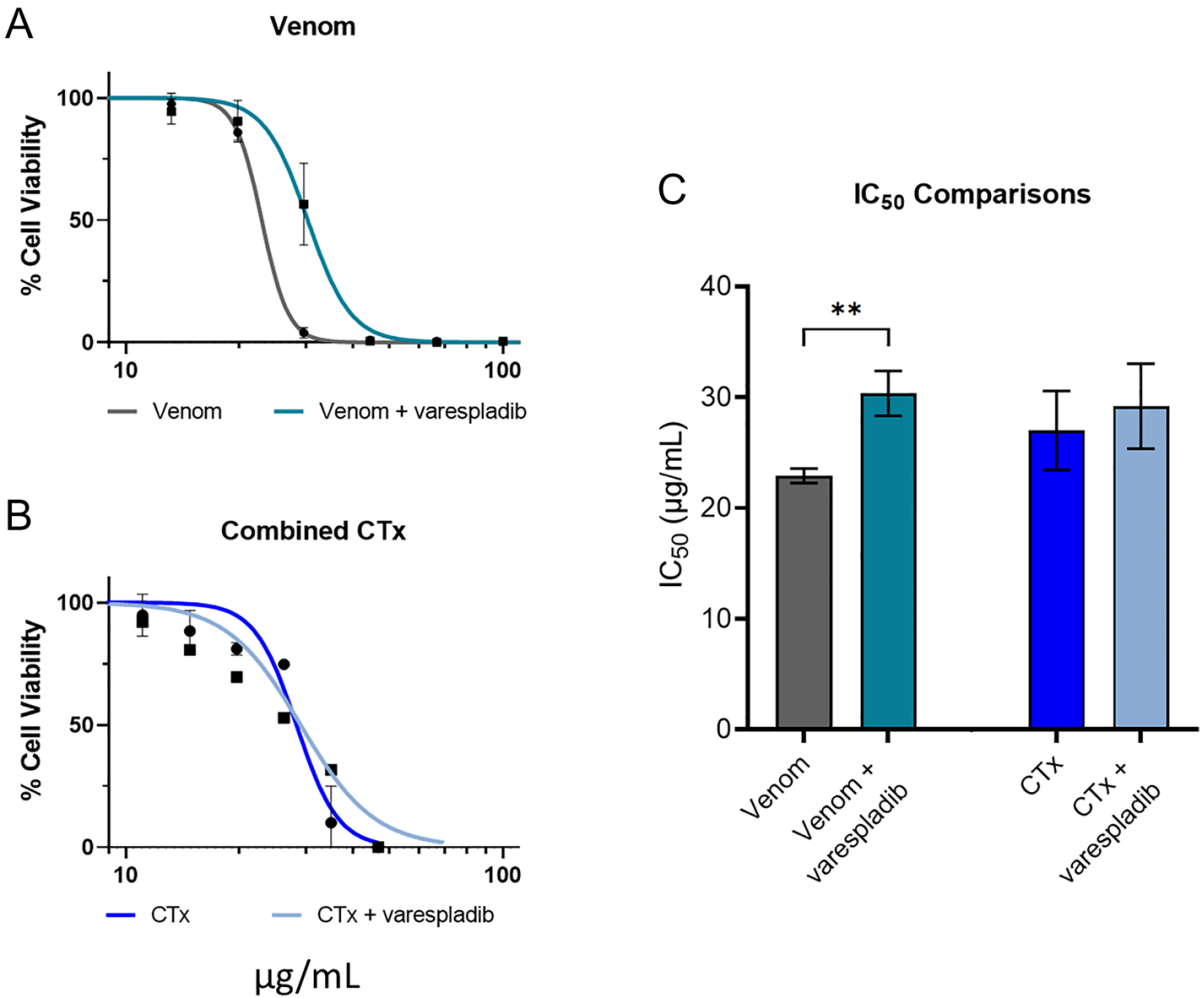
Preincubation with the PLA_2_ inhibitor varespladib has no effect on CTx-induced loss of cell viability in keratinocytes. Cell viability was measured in immortalized human keratinocytes (HaCaT cells) using MTT assays, with cells treated for 24 h with serial dilutions of (*A*) East African (Tanzania) *N. nigricollis* venom or (*B*) the purified CTx combination, with or without preincubation with 128 μM varespladib. The data shown represent mean percentage cell viability and corresponding SDs, with data normalized to 0 to 100% between the lowest and highest absorbance values for analysis, then plotted as dose–response curves using GraphPad Prism 9. All data displayed are from three independent experiments with each condition in triplicate. (*C*) IC_50_ values of the venom and CTx combination, with and without varespladib, displayed in (*A* and *B*). The data shown represent the mean IC_50_ values of curves and corresponding SDs. Statistically significant differences were determined by unpaired *t* tests and are denoted by asterisks: **P < 0.01.

To confirm that the inhibitory effect of varespladib is the sole result of inhibition of PLA_2-_driven toxicity, we performed MTT cell cytotoxicity assays, as described previously, using either East African *N. nigricollis* venom or the CTx combination preincubated with and without a cell-tolerated high dose of varespladib (128 μM) (41). As anticipated, while varespladib significantly reduced the cytotoxicity of crude venom (22.9 μg/mL ± 0.7 vs. 30.3 μg/mL ± 2.0, *P* = 0.004), no significant effect on cell viability was observed when the PLA_2_ inhibitor was coincubated with purified CTx (27.0 μg/mL ± 3.6 vs. 29.2 μg/mL ± 3.8, *P* > 0.05) (Fig. 4), thereby confirming that varespladib is only interacting with PLA_2_ toxins.

**Fig. 5.**
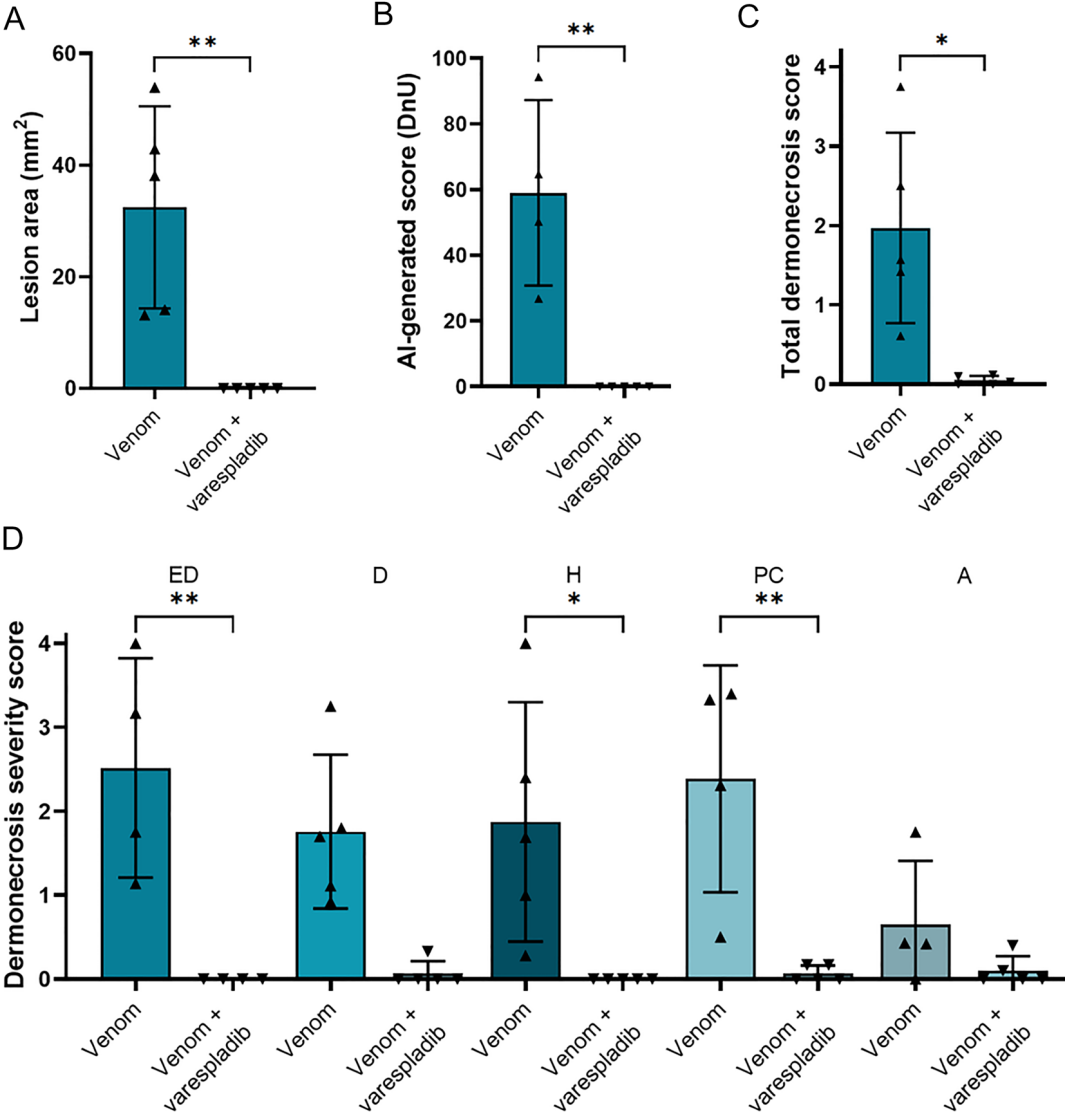
Dermonecrosis caused by *N. pallida* venom is prevented by the PLA_2_ inhibitor varespladib. Groups of mice (n = 5) were intradermally injected with 25 µg *N. pallida* venom with or without the PLA_2_ inhibiting drug varespladib (19 μg). At 72 h postinjection, lesions were excised and examined, from which it was determined that the PLA_2_ inhibitor varespladib significantly reduced the size and severity of the dermonecrotic lesions caused by *N. pallida* venom as measured with (*A*) calipers, (*B*) VIDAL, and (*C*) histopathological analysis of the (*D*) different skin layers (ED, epidermis; D, dermis; H, hypodermis; PC, panniculus carnosus; A, adventitia). For (*C* and *D*), the data shown represent the total mean dermonecrosis score of all layers, vs. the mean damage score for each individual skin layer, respectively, and corresponding SDs. Statistically significant differences were determined by unpaired *t* test comparisons for (*A–C*), and by two-way ANOVA, followed by Dunnett’s multiple comparisons tests for (*D*). Statistically significant differences are denoted by asterisks: *P < 0.05, **P < 0.01. Error bars represent SDs.

To investigate whether the inhibitory effect of varespladib might extend to other cobra species, we next used venom from a related African spitting cobra species, the red spitting cobra *N. pallida*, which diverged from *N. nigricollis* around 6.7 Mya (21), using the same in vivo model of dermonecrosis. Preincubation with varespladib resulted in complete abolition of lesion formation caused by *N. pallida* venom, with no lesions observed in any of the five experimental animals receiving the drug (venom, 32.4 mm^2^ ± 18.1 vs. venom and varespladib, 0.0 mm^2^ ± 0.0; *P* = 0.0039) (Fig. 5*A*); a result that was also confirmed with the lesion severity scores calculated by VIDAL (59.0 DnU ± 28.2 vs. 0.0 DnU ± 0.0, respectively; *P* = 0.0054) (Fig. 5*B*). Images of the resulting dermonecrotic lesions are presented in *SI Appendix*, Table S3. Further, histopathological assessment of venom-induced skin pathology also resulted in significant decreases in both total dermonecrosis scores (venom, 2.0 ± 1.2; venom and varespladib, 0.1 ± 0.1; *P* = 0.009) and for several individual skin layers analyzed (epidermis, *P* = 0.003; hypodermis, *P* = 0.027; panniculus carnosus, *P* = 0.005) (Fig. 5 *C* and *D*). Given that spitting cobra venom profiles share high levels of toxin similarity (21, 32), these findings provide confidence in the general effectiveness of varespladib against venom-induced dermonecrosis stimulated by geographically diverse African spitting cobra venoms.

### Varespladib Exhibits In Vivo Efficacy against Spitting Cobra–Induced Dermonecrosis in Delayed Treatment Models.

Next, we sought to explore the inhibitory capability of varespladib in more biologically realistic models of snakebite envenoming, where treatment is delivered after venom challenge (48, 49). In addition, to further assess the cross-species and regional efficacy of varespladib we used another venom, this time from West African (Nigerian) *N. nigricollis*.

#### Dermonecrosis: Intradermal administration of varespladib.

**Fig. 6.**
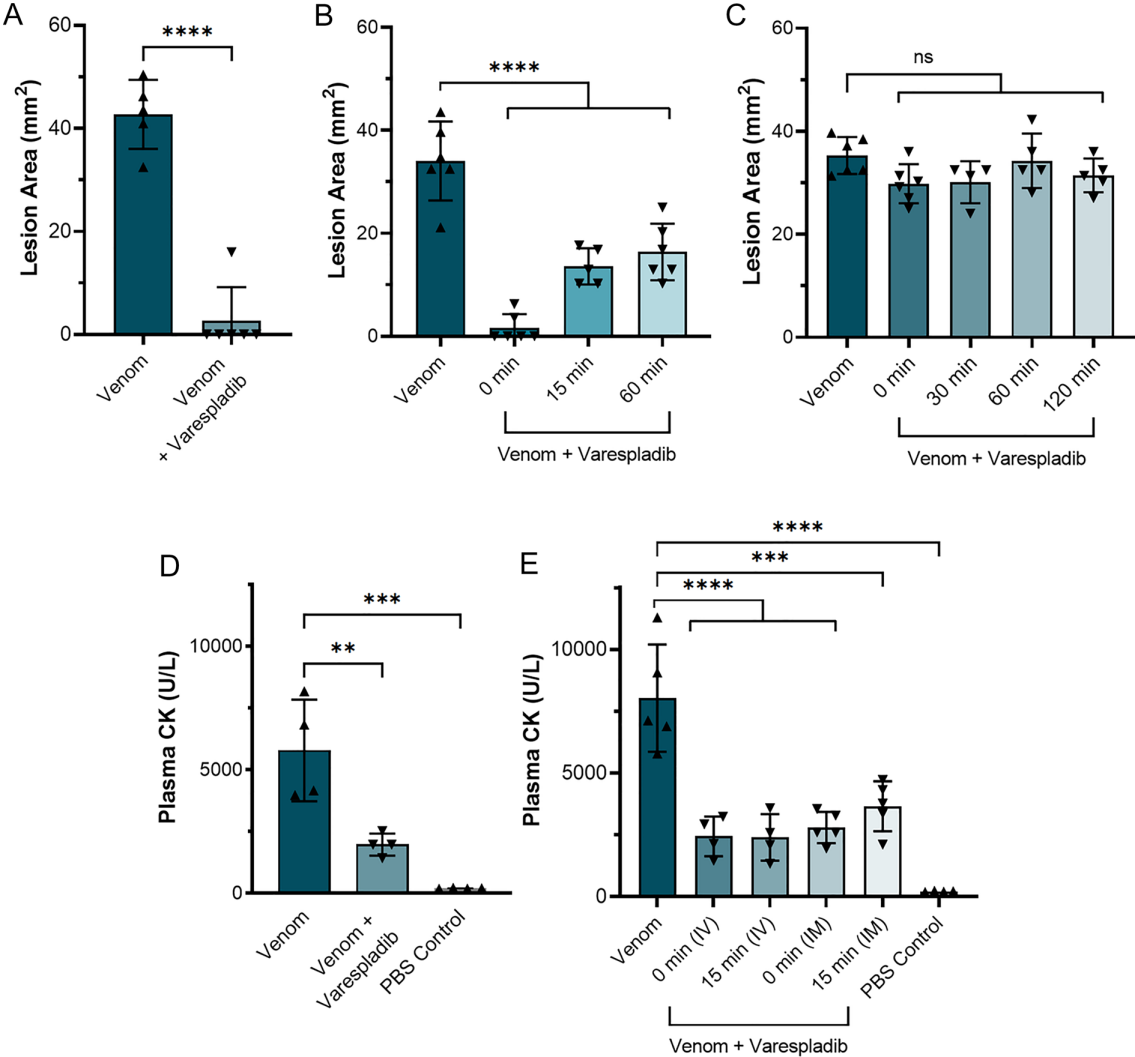
Delayed administration of varespladib following spitting cobra envenoming causes significant reductions in dermonecrosis and myotoxicity in vivo. Groups of mice (n ≥ 4) were injected with either West African (Nigeria) *N. nigricollis* venom alone or followed by varespladib at a range of different timepoints and via different administration routes. (*A*) Intradermal coadministration of preincubated venom (110 μg) and varespladib (20 µg) significantly reduced the size of skin lesions caused by West African *N. nigricollis* venom 72 h later. (*B*) Intradermal administration of varespladib (100 µg) 0, 15, and 60 min after intradermal venom challenge (110 μg) resulted in significant reductions in the size of venom-induced dermonecrotic lesions. (*C*) Intravenous delivery of varespladib (100 μg) at 0, 30, 60, and 120 min after intradermal venom challenge (110 μg) resulted in no protection against venom-induced dermonecrosis. (*D*) Intramuscular delivery of varespladib (10 µg) coincubated with 10 μg venom resulted in significant reductions in plasma CK activity induced by *N. nigricollis* venom. (*E*) Intravenous (IV) and intramuscular (IM) delivery of varespladib (100 μg) 0 or 15 min after intramuscular venom challenge (10 μg) resulted in significant reductions in plasma CK activity induced by *N. nigricollis* venom. For panel *A*, statistically significant differences were determined by an unpaired *t* test; for panels *B*–*D*, by one-way ANOVAs followed by Dunnett's multiple comparisons post hoc tests. Statistically significant differences are denoted by asterisks: *P < 0.05, ***P < 0.001, and ****P < 0.0001.

We repeated our previously described preincubation experiments and demonstrated again that the intradermal coadministration of venom with varespladib significantly reduced the size of the resulting skin lesions (42.8 mm^2^ ± 6.7 for venom vs. 2.7 mm^2^ ± 6.5 for venom and varespladib; *P* < 0.0001) (Fig. 6*A*). Then, to better understand whether varespladib could prevent dermonecrosis when administered after envenoming has occurred, and thus more accurately mimic a real-world snakebite scenario, we intradermally injected groups of mice with *N. nigricollis* venom (110 µg) followed by a second intradermal injection of varespladib (100 μg) in the same location at either 0, 15, or 60 min later. In all instances, we observed a significant reduction in the size of dermonecrotic lesions when varespladib was administered in comparison with the venom-only control (Fig. 6*B*). Reductions were most substantial in the group that received varespladib immediately after venom injection (0 min), where only one of the experimental animals presented with a small lesion 72 h later, resulting in significantly reduced mean lesion areas of 1.6 mm^2^ (±2.7) compared with 34.0 mm^2^ (±7.7) in the venom-only controls (*P* < 0.0001) (Fig. 6*B*). While we observed reduced therapeutic potency with longer time delays between venom challenge and treatment, reductions in lesion sizes remained statistically significant at both 15- and 60-min post-venom challenge (13.6 mm^2^ ± 3.5 and 16.4 mm^2^ ± 5.5, respectively, vs. 34.0 mm^2^ ± 7.7 with the venom only control; *P* < 0.0001) (Fig. 6*B*).

#### Dermonecrosis: Intravenous administration of varespladib.

To closer mimic current treatment of envenoming with antivenom, we next explored whether intravenous, rather than local, injection of varespladib could reduce venom-induced dermonecrosis. To that end, groups of experimental animals received the same intradermal dose of *N. nigricollis* venom (110 µg), followed by an intravenous injection of varespladib (100 μg) at either 0-, 30-, 60-, or 120-min post-venom challenge. However, this dose of intravenous varespladib did not provide any reduction in the size of dermonecrotic lesions when compared with the venom-only control for any of the different dosing timepoints, including when varespladib was injected immediately after the venom challenge (35.3 mm^2^ ± 3.6 for the venom only control vs. 30.1 to 34.3 mm^2^ for the various timepoint varespladib treatment groups) (Fig. 6*C*).

#### Myotoxicity: Intravenous and intramuscular administration of varespladib.

Given the promising therapeutic findings observed when using varespladib against spitting cobra venoms that cause dermonecrosis, our final experiments assessed whether varespladib could also prevent venom-induced myotoxicity—an envenoming pathology also associated with the cytotoxic action of cobra venoms in vivo (50–52) and in cultures of myogenic cell lines (16). We induced myotoxicity in our murine envenoming model via intramuscular (gastrocnemius) venom injection and quantified muscle tissue damage by measuring plasma creatine kinase (CK) (53) activity 3 h later. When West African *N. nigricollis* venom (10 µg) was coadministered with varespladib [10 µg (52)], there was a significant reduction in resulting plasma CK levels compared to those obtained from mice receiving venom alone (venom, 5783.50 U/L ± 2055.86; venom and varespladib, 1969.50 U/L ± 448.67; *P* = 0.01) (Fig. 6*D*). Following this, we investigated whether the delayed administration of varespladib would retain efficacy against venom-induced myotoxicity, and we explored this via both intramuscular and intravenous delivery of the drug at the increased dose of 100 µg, matching the route of venom challenge and current antivenom delivery, respectively. Treatment via both dosing routes resulted in significant reductions in venom-induced increases in plasma CK activity, irrespective of whether treatment was delivered immediately after venom challenge or 15 min later (54.48 to 69.59% reduction of plasma CK after varespladib injection vs. venom-only control; *P* ≤ 0.018 for all comparisons). Unlike that observed with the dermonecrosis experiments, there was little difference in drug efficacy between the two delivery routes tested, with the mean plasma CK activity of mice that received intravenous varespladib marginally lower than in those that received the therapy intramuscularly (2442.75 vs. 2797.40, 0 min; 2398.75 vs. 3656.80, 15 min; intravenous vs. intramuscular, respectively), though these differences were not statistically significant (Fig. 6*E*).

## Discussion

Snakebite results in 2.5 million envenomings each year (54), yet outdated animal-derived antivenoms remain the only specific treatment available (1). Although these therapeutics are undoubtedly lifesaving interventions, their clinical utility is restricted by a lack of affordability, limited breadth of efficacy across different snake species, high incidences of adverse reactions associated with some products, and the necessity for snakebite patients to travel, often for several hours, to a hospital for intravenous antivenom administration (1, 22). Critically, antivenoms remain ineffective against the rapidly developing local pathology of snakebite envenoming, which in severe cases can lead to patients requiring tissue debridement around the bite site or amputation of the affected limb or digit (22). There is therefore an urgent and compelling need to develop new therapies against local envenoming by African and Asian cobras (*Naja* spp.), particularly African spitting cobras (6, 55, 56). Consequently, in this study, we sought to i) characterize the dermonecrosis caused by the venoms of African spitting cobras, ii) identify the primary etiological dermonecrosis toxins found within these venoms and iii) determine whether spitting cobra venom-induced dermonecrosis can be inhibited with the PLA_2_-inhibiting drug varespladib.

Using venoms from the black-necked spitting cobra *N. nigricollis* (East and West African), we first characterized dermonecrotic pathology in a murine model of local envenoming. The results of these studies confirmed a dose-dependent relationship between the amount of venom injected and lesion severity, that the dermal lesions caused by spitting cobra venoms in vivo often contain a dark inner region surrounded by a lighter region of skin damage, and that these dark regions show more prominent microscopic damage than the lighter regions. Studies on dermonecrosis induced by cobra venoms, and their inhibition, should therefore consider this dichotomy in the assessment of the pathological effects of their venoms (40).

To elucidate the toxins responsible for inducing dermonecrosis, we first used cell cytotoxicity assays with human epidermal keratinocytes as our model, from which we demonstrated that the CTx in East African *N. nigricollis* venom are the toxins predominately responsible for in vitro cytotoxicity. Despite PLA_2_ toxins having been described as having cytotoxic effects on different cell types (18, 57), the two types of PLA_2_ isolated from *N. nigricollis* venom had little effect on keratinocytes in isolation or when combined, though the basic PLA_2_ appeared to be slightly more cytotoxic at high concentrations than the acidic PLA_2_. Crucially, the combination of purified CTx did not completely replicate the cytotoxic potency of whole venom, which instead required using both the CTx and PLA_2_ combinations together (Fig. 2*G*), suggesting that PLA_2_ toxins at least mildly potentiate the cytotoxic activity of the CTx in cell culture experiments. This probable synergy between CTx and PLA_2_ has been documented in several previous studies that explored the venom of *Naja* and related elapid snake species (19, 21, 58–61); therefore, these findings are largely consistent with the literature, although the relative contribution of CTx vs. PLA_2_-mediated cytotoxicity observed here is perhaps surprisingly skewed heavily toward CTx.

The likely synergy between the two toxin families became more apparent in vivo (Fig. 3 *A*–*C*), where both CTx and PLA_2_ in isolation were found to only cause negligible dermonecrosis in mice, while their combination resulted in extensive dermonecrotic lesions approaching the size and severity of those formed by crude venom, notwithstanding wide interanimal variability. Further, the CTx only caused modest damage to a single skin layer, the panniculus carnosus, in agreement with the known in vivo myotoxic effect of cytotoxic 3FTxs (51). Comparatively, venom and the CTx and PLA_2_ combinations together affected all skin layers, which is to be expected based on the large lesions formed by both in vivo (*SI Appendix*, Fig. S10). A prior study by Rivel et al. also found that *N. nigricollis* venom caused murine necrosis of the dermis and loss of the epidermis, though they proposed that CTx were the primary driver for this damage (62). Similar conclusions were also made by Ho et al. when investigating dermonecrosis caused by the Asian nonspitting cobra *Naja atra* (63). These findings contrast somewhat with the results of this study, which suggested that the purified CTx, which were predominately responsible for cytotoxicity in cell culture (Fig. 2), require the action of PLA_2_s to cause extensive dermonecrosis in vivo. Furthermore, histopathologically determined dermonecrosis severity scores in PLA_2_-induced lesions were high, despite the small gross lesion sizes. These data may suggest that PLA_2_ toxins can cause severe dermonecrosis focally, but without the presence of CTx this damage is limited in extent.

This necessity for both CTx and PLA_2_ toxins to be injected concurrently to cause extensive dermonecrosis is a notable finding and directly correlates with recent data demonstrating that spitting cobra PLA_2_ toxins have evolved to potentiate the algesic effect of CTx to cause enhanced pain during defensive venom spitting (21). This means that spitting cobra dermonecrosis, and thus morbidity observed in snakebite victims, may be a direct consequence of the defensive origin of cobra venom spitting. In the context of snakebite therapeutics, our findings evidencing that a combination of CTx and PLA_2_ are required to cause dermonecrosis in vivo are notable, because they suggest that inhibiting either one of these toxin families could significantly reduce the overall pathology caused by the venom.

Varespladib inhibits PLA_2_ from a range of snake venoms, including nonspitting cobras, such as *Naja naja*, *N. atra*, and *Naja kaouthia* (30), other elapids (31, 63) and several viperids (30, 31). In contrast to PLA_2_, 3FTx are poorly immunogenic due to their small size and are nonenzymatic, making them more challenging therapeutic targets (64), and to date no broadly inhibitory anti-CTx repurposed drugs have been identified. Consequently, in this study, we explored the therapeutic potential of varespladib, which has been selected as a lead candidate PLA_2_ inhibitor, and is currently undergoing clinical development for snakebite (45). However, research associated with varespladib has primarily focused on its potential utility in preventing or delaying the onset of systemic envenoming (45, 46). It has not, until now, been explored in the context of local necrosis following spitting cobra envenoming. Our in vivo preclinical efficacy experiments demonstrate that varespladib holds much therapeutic promise for this indication, as cotreatment with varespladib significantly inhibited the formation of dermal lesions caused by East and West African *N. nigricollis* and *N. pallida* venoms (Figs. 3, 5, and 6). We found no evidence that varespladib inhibits the activity of CTx (Figs. 3 and 4), and thus these data support our hypothesis that a single drug targeting one toxin family (PLA_2_) can significantly reduce the severity of local envenoming caused by cobra venom. This is particularly noteworthy when considering that *Naja* venoms typically contain more CTx than PLA_2_, often twice as much based on venom weight, and that spitting cobra venoms share a relatively high degree of compositional similarity to one another (12, 21, 32, 65), particularly in the wider context of interspecific venom variation (12, 66). Given that varespladib previously entered Phase III clinical trials for other indications (67), and that its oral prodrug form varespladib-methyl has entered Phase II clinical trials for snakebite in the USA and India (45), our findings suggest that repurposing this drug as a broad-spectrum treatment for preventing spitting cobra-induced dermonecrosis could be a valuable future application to mitigate snakebite morbidity in Africa.

Despite these exciting findings, it was important to address the limitations with the animal model of venom-induced dermonecrosis described above (49), where venom challenge and treatment are preincubated and coadministered in a manner artificial to real-world treatment of snakebite envenoming. Consequently, we assessed whether the observed efficacy of varespladib held when used as a treatment after venom challenge, for which we used West African *N. nigricollis* venom (Fig. 6). Independent intradermal injection of varespladib at the same site of the venom challenge resulted in significant reductions in the resulting venom-induced lesion sizes, even when treatment was delayed until 60 min after envenoming, and treatment with varespladib immediately after venom challenge resulted in comparable efficacy to when the drug was coadministered in the preincubation model (Fig. 6 *A* and *B*). Together, these data suggest that varespladib introduced directly into the tissue where a victim was bitten could significantly reduce the resulting dermonecrosis, particularly if administered soon after a bite. Transdermal drug delivery systems are well-established approaches that could be readily applied here to achieve rapid delivery of varespladib to snakebite victims in a community setting (68, 69). Such an approach has the potential to drastically reduce the time between bite to initial treatment from hours or days (25, 26, 70) to minutes, thus drastically improving the prognosis of tropical snakebite victims.

Since varespladib administered intravenously proved ineffective against venom-induced dermonecrosis (Fig. 6*C*), these findings suggest that an intravenous, and therefore also an oral, version of the drug is less likely to be effective at preventing dermonecrosis. However, these data may simply reflect that, at the dose tested, insufficient varespladib is able to rapidly penetrate from the circulation into the affected peripheral tissue to prevent venom toxicity. Additionally, perhaps a different venom-inhibiting molecule with superior tissue-penetrating properties to varespladib would prove more effective in such experiments. Pharmacokinetic (PK) experiments are therefore required to robustly explore whether intravenous or oral delivery of PK-optimized doses of varespladib, or other inhibitors, might also be effective routes of delivery for the treatment of severe local dermonecrosis.

The myonecrosis-reducing effects of both intramuscular and intravenous injected varespladib (Fig. 6*E*) suggest that both local and central methods of administration could be effective at preventing muscle toxicity associated with cobra snakebites. We hypothesize this difference in efficacy between myo- and dermonecrosis rescue is due to the comparatively greater abundance of blood vessels in muscle vs. cutaneous tissue, resulting in a greater and more rapid distribution of intravenously administered varespladib to the former (71, 72).

Despite the promising results of this study, there are several limitations. First, the variation between the results of our in vitro and in vivo assays demonstrates that the action of spitting cobra venoms or toxins on keratinocytes does not replicate the complex pathological effects caused in skin in vivo. This highlights the need for further research into developing more accurate in vitro models of dermonecrosis, with organoids, organotypics, and/or ex vivo skin models seeming likely to be valuable tools for future research (73). Additionally, murine models can only act as a guide for the effect a treatment may have in human patients (74), given differences between human and murine metabolism and immune systems (75), as well as differences in skin thickness and structure (76). Further, clinical trials will be needed to fully gauge the effect of varespladib against the local tissue-damaging effects of spitting cobra venoms in humans. Finally, our data were entirely focused on certain African spitting cobra venoms, specifically from two localities of *N. nigricollis* and one of *N. pallida*. Despite seemingly similar venom compositions among certain groups of cobras (21, 32), similar experiments should be performed using the venoms of other cobra species from additional localities, particularly those found in Asia, to better determine the pancobra potential of varespladib as a treatment for local envenoming and to further investigate which toxins are primarily contributing to this pathology in other settings.

In summary, our study has shown that CTx found in black-necked spitting cobra venom are largely responsible for causing venom cytotoxicity in cellular assays, but that both CTx and PLA_2_ together are required to largely recapitulate the dermonecrotic effects of crude venom in vivo. Consequently, a drug that inhibits just one of these toxin types is likely to significantly reduce the overall dermonecrosis caused by crude venom. We tested this hypothesis using the repurposed PLA_2_-inhibiting drug varespladib and demonstrated impressive preclinical efficacy of the drug against three geographically diverse African spitting cobra venoms. Most notably, the local injection of varespladib was able to significantly reduce the extent of dermonecrosis, even when dosed up to an hour after venom challenge, and protection conferred by the drug also extended to venom-induced myotoxicity. Collectively, our data suggest that varespladib could become an invaluable treatment against the tissue-damaging effects of black-necked and red spitting cobra venoms, which cause extensive morbidity in snakebite victims across the African continent.

## Materials and Methods

### Chemicals, Drugs, and Biological Materials.

See *SI Appendix*, *Methods S1* for full detail on chemicals, drugs, and biological materials used in this study.

### Venoms.

Venom pools were from wild-caught animals of differing geographical origins, namely: *N. nigricollis* (Tanzania and Nigeria; four individuals in each venom pool), and *N. pallida* (Tanzania; one individual). Venoms were sourced and stored as described in *SI Appendix*, *Methods S2*.

### Toxin Isolation.

Toxins from East African (Tanzanian) *N. nigricollis* venom were initially separated into 3FTx and PLA_2_ toxins using gel filtration chromatography (*SI Appendix*, Figs. S1–S3). Acidic PLA_2_ consisted of toxins from two individual peaks (peaks 3 and 4) which were evaluated through SDS-PAGE and RP-HPLC, with peak 3 requiring a third chromatography step for full purity (*SI Appendix*, Figs. S4 and S8 and Table S1). Basic PLA_2_ consisted of toxin from peak 11 and required dialysis before purity was confirmed through SDS-PAGE and RP-HPLC (*SI Appendix*, Figs. S5 and S8 and Table S1). The 3FTx cytotoxins were found across a range of peaks (peaks 6, 8, 9, 12 to 14) and to be varying levels of purity as determined by SDS-PAGE and RP-HPLC, with some requiring further purification steps, including trypsin digestion and MS/MS analysis (*SI Appendix*, Figs. S7–S9). See *SI Appendix*, *Methods S3* for full details of the toxin isolation methods.

### Cells.

The immortalized human epidermal keratinocyte line, HaCaT (77, 78), was purchased from Caltag Medsystems (Buckingham, UK). Cells were cultured in phenol red-containing DMEM with GlutaMAX supplemented with 10% FBS, 100 IU/mL penicillin, 250 µg/mL streptomycin, and 2 mM sodium pyruvate (standard medium; Gibco), per Caltag’s HaCaT protocol. For cell assays that contained the fluorescent dye PI, a medium formulated for fluorescence-based cell assays was used: FluoroBrite DMEM supplemented with 1% GlutaMAX 100× supplement, 1% FBS, 100 IU/mL penicillin, 250 µg/mL streptomycin, and 2 mM sodium pyruvate (minimally fluorescent medium; Gibco). The cells were split and growth medium changed 2× per week up to a maximum of 30 passages. Cells were maintained in a humidified, 95% air/5% CO_2_ atmosphere at 37 °C (standard conditions).

### Multiplexed MTT Cell Viability and PI Cell Death Assays.

MTT cell viability (34) and PI cell death assays were completed as described previously (41), with minor modifications. Briefly, *Day 1*: HaCaT cells were seeded (20,000/well). *Day 2*: Cells treated with venoms, purified toxins, or controls for 24 h. *Day 3*: PI fluorescence was read (Ex_544_/Em_612_) to measure cell death, followed by MTT assays (A_550_) to measure cell viability. See *SI Appendix*, *Methods S4* for full details.

### Animal Ethics and Maintenance.

Animal experiment protocols were performed in accordance with ethical approvals from relevant bodies, using male SWISS (CD1) mice (18 to 27 g) housed in accordance with animal welfare standards. See *SI Appendix*, *Methods S5* for full details on ethics and animal maintenance.

### In Vivo Dermonecrosis and Cotreatment with Varespladib Using a Preincubation Model of Envenoming.

Briefly, mice were intradermally injected with venoms preincubated with varespladib or control dissolved in 50 µL of PBS. After 72 h, experimental animals were humanely killed via inhalational CO_2_, and skins around the injection site dissected. Size of internal lesions was measured, and photos and excised cross-sections taken for further AI and histopathology analysis, respectively. Full details of specific treatments can be found in *SI Appendix*, *Methods S6*.

### Delayed Treatment Models of In Vivo Dermonecrosis with Varespladib.

To assess the ability of varespladib to inhibit dermonecrosis in a challenge-then-treat model, mice were first intradermally injected with venom from West African (Nigeria) *N. nigricollis*, followed by either intradermal or intravenous varespladib (or vehicle control) injection at various time points postenvenoming. At 72 h, mice were humanely killed by CO_2_ inhalation, the skin around the injection sites dissected, and lesion sizes measured. Full details can be found in *SI Appendix*, *Methods S7*.

### In Vivo Models of Myotoxicity and Treatment with Varespladib.

Briefly, mice were intramuscularly injected in the right gastrocnemius with West African (Nigeria) *N. nigricollis* venom preincubated with venom or control dissolved in 50 µL of PBS. Three hours after the envenoming event, mice were humanely killed, blood samples collected and plasma isolated, from which CK activity was quantified as a measure of muscle damage. Challenge-then-treat experiments were then completed where varespladib or control was administered either intramuscularly or intravenously at various time points postenvenoming, followed by plasma-CK quantification. Full details can be found in *SI Appendix*, *Methods S8*.

### Lesion Severity Scoring Using the Venom-Induced Dermonecrosis Analysis Tool: VIDAL.

The severity of the dermonecrotic lesions was assessed using our AI analyzer, VIDAL, the details of which can be found in Laprade et al. (40). For full details, see *SI Appendix*, *Methods S9*.

### Histopathological Analysis of Excised Tissue Samples.

Formalin-stored tissue samples were processed and embedded in paraffin, before four micrometer paraffin sections were cut and placed on color slides or poly-lysine slides to dry. The slides were hematoxylin & eosin stained and cover slipped using DPX. Brightfield images of the H&E-stained lesions were captured with an Echo Revolve microscope, and evidence of necrosis was assessed separately for the epidermis, dermis, hypodermis, panniculus carnosus, and adventitia layers, as described by Hall et al. (41). The % necrosis of each skin layer within each image was assessed by two independent and blinded pathologists and scored, with mean scores for each layer on each image determined. The “dermonecrosis severity score” was determined for each lesion by taking the mean of the individual layer scores. See *SI Appendix*, *Methods S10* for full methods on the histopathological analysis. Full histopathology image data have been deposited in FigShare (79).

### Statistical Analysis.

All data are presented as mean average ± SD of at least three independent experimental replicates. Appropriate statistical tests and post hoc tests were carried out and a difference was considered statistically significant where *P* ≤ 0.05. See *SI Appendix*, *Methods S11* for full details on the statistical analysis.

## Supplementary Material

Appendix 01 (PDF)

Dataset S01 (XLSX)

Dataset S02 (XLSX)

Dataset S03 (XLSX)

Dataset S04 (XLSX)

Dataset S05 (XLSX)

Dataset S06 (XLSX)

Dataset S07 (XLSX)

## Data Availability

Histopathology image data have been deposited in FigShare (79).
