# Supplementary material for: Dermonecrosis caused by a spitting cobra snakebite results from toxin potentiation and is prevented by the repurposed drug varespladib
Source: Proc Natl Acad Sci U S A. 2024 Apr 30;121(19):e2315597121. doi: 10.1073/pnas.2315597121 (PMC11087757; doi:10.1073/pnas.2315597121)
Supplement: Supplementary file 1 — Appendix 01 (PDF) [file pnas.2315597121.sapp.pdf]

PNAS

Supporting Information for

Dermonecrosis caused by a spitting cobra snakebite results from toxin potentiation and is prevented by the repurposed drug varespladib

Keirah E. Bartlett[1†], Steven R. Hall[1,2†], Sean A. Rasmussen[3], Edouard Crittenden[1], Charlotte A. Dawson[1], Laura-Oana Albulescu[1,2], William Laprade[4], Robert A. Harrison[1,2], Anthony J. Saviola[5], Cassandra M. Modahl[1], Timothy P. Jenkins[6], Mark C. Wilkinson[1], José María Gutiérrez[7], Nicholas R. Casewell[1,2*]

[1]Centre for Snakebite Research & Interventions, Department of Tropical Disease Biology, Liverpool School of Tropical Medicine, Pembroke Place, Liverpool, L3 5QA, United Kingdom.

[2]Centre for Drugs & Diagnostics, Liverpool School of Tropical Medicine, Pembroke Place, Liverpool, L3 5QA, United Kingdom.

[3]Department of Pathology and Laboratory Medicine, Queen Elizabeth II Health Sciences Centre and Dalhousie University, 7th Floor of MacKenzie Building, 5788 University Avenue, Halifax, Nova Scotia, B3H 1V8, Canada.

[4]Department of Applied Mathematics and Computer Science, Technical University of Denmark, Kongens Lyngby, Denmark

[5]Department of USA Biochemistry and Molecular Genetics, 12801 East 17th Avenue, University of Colorado Denver, Aurora, CO 80045, USA

[6]Department of Biotechnology and Biomedicine, Technical University of Denmark, Kongens Lyngby, Denmark

[7]Instituto Clodomiro Picado, Facultad de Microbiología, Universidad de Costa Rica, PO Box 11501-2060, San José, Costa Rica.

[†] These authors contributed equally
* Corresponding author: Nicholas R. Casewell
Email: nicholas.casewell@lstmed.ac.uk

This PDF file includes:

Methods S1 to S7
Figures S1 to S11
Tables S1 to S3
Legend for Datasets S1-S7

Other supporting materials for this manuscript include the following:

Datasets S1-S7

Methods S1: Chemicals, Drugs and Biological Materials

Thiazolyl blue methyltetrazolium bromide (MTT; M5655), dimethyl sulfoxide (DMSO; 276855), propidium iodide (PI; P4170), and varespladib (SML1100; stock solution of 65.7 mM [25 mg/mL] in DMSO) were purchased from Sigma-Aldrich (Merck). Varespladib used in delayed treatment *in vivo* experiments was provided by Dr. Matthew Lewin (Ophirex Inc., Corte Madera, CA, USA). Dulbecco's modified Eagle's medium (DMEM; 11574516), foetal bovine serum (FBS; 11573397), FluoroBrite DMEM (A1896701), glutaMAX supplement (35050038), penicillin-streptomycin (11528876), phosphate buffered saline (11503387), and TrypLE Express were purchased from Gibco (Thermo Fisher Scientific).

Methods S2: Venoms

Venoms were sourced from snake specimens maintained in the Liverpool School of Tropical Medicine's (LSTM) Herpetarium. This facility and its snake husbandry protocols are approved and inspected by the UK Home Office and the LSTM Animal Welfare and Ethical Review Boards. Venom pools were from wild-caught animals of differing geographical origins, namely: *Naja nigricollis* (Tanzania and Nigeria; four individuals in each venom pool), and *N. pallida* (Tanzania; one individual). Crude venoms were lyophilised and stored at 4 °C to ensure long-term stability, then resuspended in PBS (10 mg/mL) and kept at -80 °C until used in the described experiments, with freeze-thaw cycles kept to a minimum to prevent degradation.

Methods S3: Toxin isolation

The initial step in toxin isolation was a large-scale size separation on a gel filtration chromatography column; this separates the 3FTx and PLA_2 toxins from the higher molecular weight proteins (i.e. snake venom metalloproteinases, L-amino acid oxidase and cysteine rich secretory proteins). For this, 100 mg of *N. nigricollis* (Tanzania) venom was dissolved in 5 mL ice-cold PBS (25 mM sodium phosphate pH 7.2, 0.15 M NaCl) and centrifuged at 10,000 xg for 10 minutes. The supernatant was immediately loaded onto a 480 mL column (2.6 x 95 cm) of Superdex 200HR equilibrated in PBS. The column was operated at a flow rate of 1.0 mL/min during loading and 2.0 mL/min for elution. 10 mL fractions were collected after the void volume and elution was monitored at 280 nm (see **Fig. S1**). SDS-PAGE analysis was carried out on all fractions seen to contain protein on the trace. PLA_2 and 3FTx, respectively, were found to be wholly contained in overlapping peaks 1 and 2. Since their separation was incomplete at this stage, these peaks were pooled in preparation for a second step on cation exchange chromatography. This PLA_2/3FTx pool was dialysed against 50 mM sodium phosphate, pH 6.0 and then applied to a 20 mL HPSP HiPrep column equilibrated in the same buffer. Elution was carried out using a 15-column volume (CV) gradient of 0 – 0.7 M NaCl in 50 mM sodium phosphate, pH 6.0. The flow rate was 1.5 mL/min. The unbound material was retained and 10 mL fractions collected from the start of the NaCl gradient. Elution was monitored at 280 nm (**Fig. S2**). SDS-PAGE analysis was carried out on the main peaks (**Fig. S3**). Cation exchange peaks 3, 4, and 11 contained a band at 14 kDa, assumed to be PLA_2 and based on the relative elution position, 3, 4 are acidic PLA_2 and 11 is basic PLA_2. Peaks 5-9 each contained equal-sized bands that ran just below the 10 kDa marker, characteristic of 3FTx proteins. The major 3FTx fractions, peaks 6, 8, and 9, were pooled back together and dialysed against PBS to form the whole 3FTx fraction, containing the major 3FTx cytotoxins 1, 3 and 4 [see below], used for the *in vivo* studies, and referred to as CTx in this study.

Purification of acidic PLA_2. As judged by SDS-PAGE and RP-HPLC (see **Fig. S8**), the PLA_2 in peak 4 was sufficiently pure for cytotoxicity studies. It was found to have a mass of 13,287 Da. MS/MS of tryptic peptides (see methods below) matched this protein to acidic phospholipase A2 CM-I P00602 from *N. mossambica* (see **Table S1**). The PLA_2 in peak 3 required a third chromatography step for full purity. This fraction was loaded directly onto a 4.7 mL column of Phenyl Sepharose LS FF equilibrated in 25 mM sodium phosphate pH 7.2. Elution was carried out with a 0-100% (4 CV) gradient of 25 mM sodium phosphate pH 7.2 to 25% ethanol in 25 mM sodium phosphate pH 7.2 with long hold step (5 CV) in the latter buffer. The column was operated at 1.0 mL/min and elution was monitored at 214 nm (see **Fig. S4**). The mass of the purified protein, eluting between 30 and 40 minutes, was found to be 13,172 Da and it was also matched to acidic phospholipase A2 CM-I P00602 ('acidic PLA_2') from *N. mossambica* following trypsin digestion and MS/MS (see **Table S1**). The two acidic PLA_2 forms were combined and used as a single acidic PLA_2 pool throughout the study.

Purification of basic PLA_2. Peak 11 from the cation exchange chromatography step was dialysed against 5 mM sodium phosphate pH 6.8 and then loaded onto a 1 mL column of ceramic hydroxyapatite (CHT I, BioRad) equilibrated in the same buffer. Using a flow rate of 0.5 mL/min, elution was carried out with gradient of 5 mM sodium phosphate pH 6.8 to 500 mM sodium phosphate pH 6.8, 0.15 M NaCl over 20 CV. Elution was monitored at 214 nm (see **Fig. S5**). The protein in the main peak (eluting between 35 and 40 mins) was pure as determined by SDS-PAGE and RP-HPLC (see **Fig. S8**), and was found to have a mass of 13,249 Da. Trypsin digestion with MS/MS analysis resulted in a match to Phospholipase A2 'basic' P00605 ('basic PLA_2') from *N. nigricollis* (see **Table S1**).

The basic PLA_2 and the two acidic PLA_2 were pooled back together and dialysed against PBS to form the combined PLA_2 fraction used in the *in vivo* studies.

Purification of individual 3FTx cytotoxins for cell cytotoxicity studies. The material in peak 6 from cation exchange chromatography was fairly pure as determined by RP-HPLC, but it was highly purified using hydrophobic interaction chromatography (HIC). For this, the fraction was made up to 1.5 M in NaCl and loaded onto a 10 mL Phenyl Superose column. Proteins were then eluted in a 5 CV gradient of 1.5 M NaCl in 25 mM sodium phosphate pH 7.2 to 30% (v/v) ethylene glycol in 25 mM sodium phosphate pH 7.2. The flow rate was 1 mL/min and elution was monitored at 214 nm (see **Fig. S6**). The protein in the main peak (eluting at 50 mins) was found to be pure by SDS-PAGE and RP-HPLC and its mass was determined to be 6,817 Da. MS/MS of tryptic peptides confirmed this protein to be Cytotoxin 1 P01468, referred to as CTx1 throughout this study.

RP-HPLC analysis showed that peaks 8 and 9 together contained three main 3FTx forms. These were separated using HIC as for peak 6. The conditions were identical, except that the starting buffer was 1.2 M NaCl rather than 1.5 M. As can be seen in **Fig. S7**, the three proteins are fully separated by these means. RP-HPLC analysis showed each of these to be pure (**Fig. S9**) and to possess very characteristic peak shapes. All three were subjected to digestion with trypsin and identified using MS/MS. Peak 12 had a mass of 6,707 Da and was identified to be Cytotoxin 4 P01452 (CTx4 in this study). Peak 13 had a mass of 6,817 Da and was identified using MS/MS to be Cytotoxin 1 P01468. This is likely to be a version of the protein identified in peak 6 with a Asn/Asp modification and is named hereafter as CTx1v. Peak 14 had a mass of 6,884 Da and was determined to be Naniproin/Cytotoxin 3 P0DSN1 (CTx3).

Purity analysis methods. SDS-PAGE was carried out using 4-20% acrylamide gels (BioRad) and run using a Tris-glycine buffer system, followed by staining with Coomassie Blue R250. RP-HPLC was performed on a Vanquish HPLC system (Thermo) using a Biobasic C4 column (2.1 x 150 mm). The flow rate was 0.2 mL/min and proteins were separated in a gradient of acetonitrile (see **Fig. S8** and **S9** for gradient formats) in 0.1% trifluoroacetic acid, with monitoring at 214 nm. Between 2-5 µg protein was typically loaded per analysis.

Trypsin digestion and MS/MS analysis. In preparation for analysis, the relevant proteins were desalted on a RP-HPLC column, as above. These were then dried in a centrifugal evaporator, 20 µL H_2O was added and then re-dried. The proteins were then resuspended in 8 M urea/0.1 M Tris-Cl (pH 8.5), reduced with 5 mM TCEP (tris (2-carboxyethyl) phosphine) for 20 min and alkylated with 50 mM 2-chloroacetamide for 15 min in the dark at room temperature. Samples were diluted 4-fold with 100 mM Tris-Cl (pH 8.5) and digested with trypsin at an enzyme/substrate ratio of 1:20 overnight at 37°C. The reaction was terminated by addition of formic acid (FA), and digested peptides were loaded on to Evotips and analysed directly using an Evosep One liquid chromatography system (Evosep Biosystems, Denmark) coupled with timsTOF SCP-mass spectrometer (Bruker, Germany). Peptides were separated on a 75 µm i.d. × 15 cm separation column packed with 1.9 µm C18 beads (Evosep Biosystems, Denmark) and over a predetermined 44-minute gradient. Buffer A was 0.1% FA in water and buffer B was 0.1% FA in acetonitrile. Instrument control and data acquisition were performed using Compass Hystar (version 6.0) with the timsTOF SCP operating in data-dependent acquisition mode. Fragmentation spectra were searched against an in-house venom gland-derived protein sequence database using Mascot (1, 2). Reverse decoys and contaminants were included in the search database. Cysteine carbamidomethylation was selected as a fixed modification, and oxidation of methionine was selected as a variable modification. The precursor-ion mass tolerance and fragment-ion mass tolerance were set at 10 ppm and 0.04 Da, respectively, and up to two missed tryptic cleavages were allowed. Mascot files were parsed into Scaffold (version 5.0.1, Proteome Software, Inc.) for validation at a protein-level false discovery rate (FDR) of <1%.

Methods S4: Multiplexed MTT Cell Viability and PI Cell Death Assays

The MTT cell viability (3) and PI cell death assays were completed as described previously (4), with minor modifications. Briefly, HaCaT cells were seeded at 20,000 cells/well in black-sided/clear-bottomed 96-well plates in standard medium and incubated overnight in standard conditions. The following day, standard medium was aspirated and replaced with treatment solutions prepared in minimally fluorescent medium supplemented with 50 µg/mL PI: wells were treated in triplicate with 100 µL of crude venom (9.09-150.00 µg/mL), individual purified CTx (9.09-150.00 µg/mL), individual purified PLA_2 (9.09-150.00 µg/mL), pooled purified CTx (5.96-44.67 µg/mL), pooled purified PLA_2 (0.0005-500 µg/mL), or pooled purified CTx + PLA_2 in a 2:1 ratio (4.68-35 µg/mL) sourced from East African (Tanzanian) *N. nigricollis* venom, then placed back in standard conditions for a further 24 hours. PI fluorescent readings (Ex_{544}/Em_{612}) were collected on a CLARIOstar Plus (BMG Labtech). The treatment solutions were then aspirated and replaced with MTT-containing minimally fluorescent medium (120 µL at 0.83 mg/mL), and the plates incubated for 45 min in standard conditions. Thereafter, the MTT-containing medium was aspirated, 100 µL DMSO added to each well to dissolve the formazan crystals, and absorbance (550 nm) was read for all wells on the CLARIOstar. Experiments were repeated independently on three occasions for each venom or toxin. Subsequently data were normalised to 0-100% between the lowest and highest absorbance values for analysis to represent %-cell death (PI) or %-cell

viability (MTT), then plotted as dose-response curves using GraphPad Prism 9. IC_{50} (MTT) and EC_{50} (PI) values were calculated using the '[Inhibitor] vs. normalized response – Variable slope' and '[Agonist] vs. normalized response – Variable slope' functions, respectively.

Methods S5: Animal ethics and maintenance

Animal experiment protocols were performed using approvals from the Animal Welfare and Ethical Review Boards of the Liverpool School of Tropical Medicine and the University of Liverpool, with licensed approval (PPL# P58464F90) from the UK Home Office, and in accordance with the UK Animal (Scientific Procedures) Act 1986, and the Institutional Committee for the Care and Use of Laboratory Animals (CICUA) of the University of Costa Rica (approval number CICUA 82-2). Male SWISS (CD1) mice (18-27 g; Charles River, Janvier, Animal Facility of Instituto Clodomiro Picado, Costa Rica) were housed in groups of five and acclimated for one week prior to experimentation. Mice used in experiments conducted in Liverpool (**Fig. 3-5**) were given *ad libitum* access to CRM irradiated food (Special Diet Services, UK) and reverse osmosis water in an automatic water system and kept under room conditions of approximately 22 °C, 40–50% humidity, with 12/12-hour light cycles. Mice used in experiments conducted in Costa Rica (**Fig. 1, 6**) were maintained under conditions of 22-24 °C and 60-65% humidity, with 12/12-hour light cycles, and given *ad libitum* access to food and water.

Methods S6: *In vivo* dermonecrosis and co-treatment with varespladib using a preincubation model of envenoming

For initial experiments (**Fig. 1**) groups of mice (18-20 g; n=3) received intradermal injections in the ventral abdominal region of either 75 µg or 100 µg of the venoms of West African (Nigeria) or East African (Tanzania) *N. nigricollis*, dissolved in 50 µL of PBS; control mice were injected with 50 µL of PBS alone. After 72 h, mice were sacrificed by CO_2 inhalation, the skins were removed, and the areas of the lesions on the inner side of the skin were measured. Then, skin samples were added to 3.7% formalin fixative solution and processed for embedding in paraffin. Sections (4 µm) were collected and stained with haematoxylin-eosin for microscopic assessment. To identify the toxins responsible for causing dermonecrosis and to assess whether varespladib inhibited this effect (**Figs. 3-5, 6A**), groups of mice (n=4-8) were briefly anaesthetised using inhalational isoflurane and then ID-injected in the rear flank with a 50 µL solution of either: (i) East African *N. nigricollis* venom (63 µg), (ii) corresponding proportional amounts of venom CTx (37.8 µg) or PLA_2 (16.4 µg) isolated from the crude venom, (iii) these purified CTx and PLA_2 combined in a 2:1 ratio, reflecting their relative abundance in crude venom (37.8 µg and 16.4 ug, respectively) (5, 6, 7), (iv) *N. pallida* (Tanzania) venom (25 µg), or (v) West African *N. nigricollis* venom (110 µg), all diluted in PBS (pH 7.4). The same experimental design was used for the varespladib-inhibition experiments except that every venom challenge dose was co-incubated with 19 µg of varespladib (4) (**Figs. 3-5**) or 20 µg of varespladib (**Fig. 6A**) (diluted in 98.48% pH 7.4 PBS and 1.52% DMSO) for 30 minutes at 37 °C and then kept on ice until shortly before intradermal injection. Following dosing, all experimental animals were observed frequently to ensure that no signs of systemic envenoming presented (e.g., neuromuscular paralysis), and the development of local lesions were monitored for 72 hrs. Thereafter, experimental animals were humanely euthanised via inhalational CO_2, and the skins around the injection site dissected. The height and width of the lesions on the inner side of the skin were measured in two directions with digital calipers, placed on A4 printout sheets to standardise the AI-lesion analyser which are

available in Jenkins, *et al.* (8), and photographed using a Sony DSC-W800 camera. Excised lesions were cut into cross-sections down the centre of the lesions, placed in plastic tissue cassettes (Sigma-Aldrich [Merck]; Z672122) and fixed in 3.7% formalin pots (CellPath; 13191184) for a maximum of 72 h prior to preparation for downstream histopathology.

Methods S7: Delayed treatment models of *in vivo* dermonecrosis with varespladib
To assess inhibition of dermonecrosis in a challenge-then-treat model (**Fig. 6**), groups of mice (n=4-6; 22-24 g) received an intradermal injection of 110 µg West African (Nigeria) *N. nigricollis* venom dissolved in 50 µL PBS. Then, at various time intervals after venom (either immediately [0 min], 15 min, or 60 min), a solution of varespladib (100 µg dissolved in 50 µL PBS) was injected intradermally at the same site of venom injection. In the case of venom only controls, 50 µL of PBS was administered intradermally at the site of venom injection immediately after venom. Similar experiments were then performed using intravenous delivery of varespladib. After the intradermal injection of 110 µg *N. nigricollis* venom, varespladib (100 µg in 50 µL PBS) was administered intravenously in the caudal vein, either immediately (0 min), or 30, 60 or 120 min after venom. After intradermal venom injection, control groups of mice received PBS intravenously instead of varespladib. At 72 h, mice were sacrificed by CO_2 inhalation, the skin was removed and the areas of the necrotic lesions in the inner side of the skin were measured as previously described.

Methods S8: *In vivo* models of myotoxicity and treatment with varespladib
To assess inhibition of myotoxicity (**Fig. 6**), West African (Nigeria) *N. nigricollis* venom was incubated with varespladib at 37 °C for 30 minutes. Then, aliquots of 50 µL, each containing 10 µg venom and 10 µg varespladib, were injected intramuscularly into the right gastrocnemius muscle of groups of mice (n=4-5; 18-20 g). Controls included mice receiving 10 µg of venom alone and mice receiving 50 µL PBS alone. Three hours after venom injection, blood samples were collected into heparinised capillary tubes under light inhaled isoflurane anaesthesia by cutting the tip of the tail. After centrifugation, the CK activity of plasma was quantified by using a commercial kit (CK-NAC-UVAA kit; Wiener Laboratories, Rosario, Argentina). CK activity was expressed as units/litre (U/L). Additionally, experiments in which varespladib was administered after venom injection were performed (**Fig. 6**). For this, mice received an intramuscular injection, in the right gastrocnemius, of 10 µg West African (Nigeria) *N. nigricollis* venom, dissolved in 50 µL PBS. At various time intervals after venom injection (either immediately [0 min], or at 15 min), a dose of 100 µg varespladib, dissolved in 50 µL PBS, was administered either intramuscularly at the site of venom injection, or intravenously in the caudal vein. A control group of mice received 10 µg venom intramuscularly and PBS by either route instead of varespladib immediately after venom injection. Another control group received only 50 µL PBS by the intramuscular route.

Methods S9: Lesion severity scoring using the Venom Induced Dermonecrosis Analysis tool: VIDAL
The severity of the dermonecrotic lesions was assessed using our newly developed AI analyser, VIDAL, the details of which can be found in Laprade, *et al* (9). Briefly, images of the dissected lesions on A4 standardisation cut out masks (8) were uploaded to the AI's website

(https://github.com/laprade117/VIDAL-Experiments). The program measured and scored the dark and light regions of the lesions from which it calculated a total dermonecrosis score, given the appropriately named Dermonecrotic Units (DnU), where the higher the number the more severe the lesion.

Methods S10: Histopathological analysis of excised tissue samples
Formalin-stored tissue samples were processed using a Tissue-Tek VIP (vacuum infiltration processor) overnight, before being embedded in paraffin (Ultraplast premium embedding medium, Solmedia, WAX060). Four micrometer paraffin sections were cut on a Leica RM2125 RT microtome, floated on a water bath, and placed on colour slides (Solmedia, MSS54511YW) or poly-lysine slides (Solmedia MSS61012S) to dry. For haematoxylin & eosin (H&E) staining, slides were dewaxed in xylene and rehydrated through descending grades of ethanol (100%, 96%, 85%, 70%) to distilled water before being stained in haematoxylin (5 mins), "blued" in tap water (5 mins) and stained in eosin (2 mins). Slides were then dehydrated through 96% and 100% ethanol to xylene and cover slipped using DPX (CellPath SEA-1304-00A). Haematoxylin (Atom Scientific, RRBD61-X) and eosin (TCS, HS250) solutions were created in house. Brightfield images of the H&E-stained lesions were captured with an Echo Revolve microscope (Settings: 10x magnification; LED: 100%; Brightness: 30; Contrast: 50; Colour balance: 50), with at least five images taken per slide. As described by Hall *et al*. (4), histological evidence of necrosis was assessed separately for the epidermis, dermis, hypodermis, panniculus carnosus, and adventitia layers. Features of necrosis included loss of nuclei, nuclear fragmentation (karyorrhexis), nuclear shrinkage and hyperchromasia (pyknosis), loss of cytoplasmic detail with hypereosinophilia, loss of cell borders and, in the case of severe necrosis, disarray with complete loss of architecture and hyalinization. In the epidermis, ulceration with superficial debris was interpreted as evidence of necrosis. In the dermis, loss of skin adnexal structures (e.g., hair follicles and sebaceous glands) and extracellular matrix disarray were also interpreted as evidence of necrosis. The %-necrosis of each skin layer (epidermis, dermis, hypodermis, panniculus carnosus, and adventitia) within each image was assessed by two independent and blinded pathologists and scored between 0 and 4, with 0 meaning no observable necrosis in the layer within that image, 1 meaning up to 25% of the layer in that image exhibiting signs of necrosis, 2 meaning 25-50% necrosis, 3 meaning 50-75%, and 4 meaning more than 75% exhibiting indicators of necrosis. The mean scores of the pathologists for each layer from each image were determined, and the highest scores-per-mouse used for our data analysis as these represented the maximum necrotic severity within each lesion. The 'dermonecrosis severity score' was determined for each lesion by taking the mean of the individual layer scores.

Methods S11: Statistical analysis
All data are presented as mean average ± standard deviation of at least three independent experimental replicates. For cell experiments, 'n' is defined as an independent experiment completed at a separate time from other 'n's within that group of experiments; all drug and/or venom treatments within an 'n' were completed in triplicate wells and the mean taken as the final value for that one trial. For *in vivo* experiments, 'n' is defined as the number of mice in that specific treatment group. Two-tailed t-tests were performed for dual comparisons, one-way analysis of variances (ANOVAs) performed for multiple comparisons with one independent variable followed by Dunnett's or Tukey's multiple comparisons tests when the trial data were

compared to a single control group or to all other groups, respectively, as recommended by GraphPad Prism, and two-way ANOVAs performed for multiple comparisons with two independent variables followed by Dunnett's multiple comparisons tests. A difference was considered statistically significant where $P \leq 0.05$.

References for Supplementary Methods

1. T. D. Kazandjian et al., Convergent evolution of pain-inducing defensive venom components in spitting cobras. *Science* **371**(6527), 386-390 (2021).

2. D. N. Perkins, D. J. Pappin, D. M. Creasy, J. S. Cottrell, Probability-based protein identification by searching sequence databases using mass spectrometry data. *Electrophoresis: An International Journal* **20**, 3551-3567 (1999).

3. T. Mosmann, Rapid colorimetric assay for cellular growth and survival: application to proliferation and cytotoxicity assays. *Journal of Immunological Methods* **65**, 55-63 (1983).

4. S. R. Hall *et al.*, Repurposed drugs and their combinations prevent morbidity-inducing dermonecrosis caused by diverse cytotoxic snake venoms. *Nature Communications* **14**(1), 7812 (2023).

5. I. Méndez , J. M. Gutiérrez , A. Angulo , J. J. Calvete , B. Lomonte, Comparative study of the cytolytic activity of snake venoms from African spitting cobras (Naja spp., Elapidae) and its neutralization by a polyspecific antivenom. *Toxicon* **58**, 558-564 (2011).

6. D. Petras et al., Snake venomics of African spitting cobras: toxin composition and assessment of congeneric cross-Reactivity of the pan-African EchiTAb-Plus-ICP antivenom by antivenomics and neutralization approaches. *Journal of Proteome Research* **10**, 1266–1280 (2011).

7. A. Sánchez et al., Comparative venomics and preclinical efficacy evaluation of a monospecific *Hemachatus* antivenom towards sub-Saharan Africa cobra venoms. *Journal of Proteomics* **240**, 104196 (2021).

8. T. P. Jenkins *et al.*, AHA: AI-guided tool for the quantification of venom-induced haemorrhage in mice. *Frontiers in Tropical Diseases* **3**, 1063640 (2022).

9. W. Laprade *et al.*, Machine-learning guided Venom Induced Dermonecrosis Analysis tooL: VIDAL. *Scientific Reports* **13**(1), 21662 (2023).

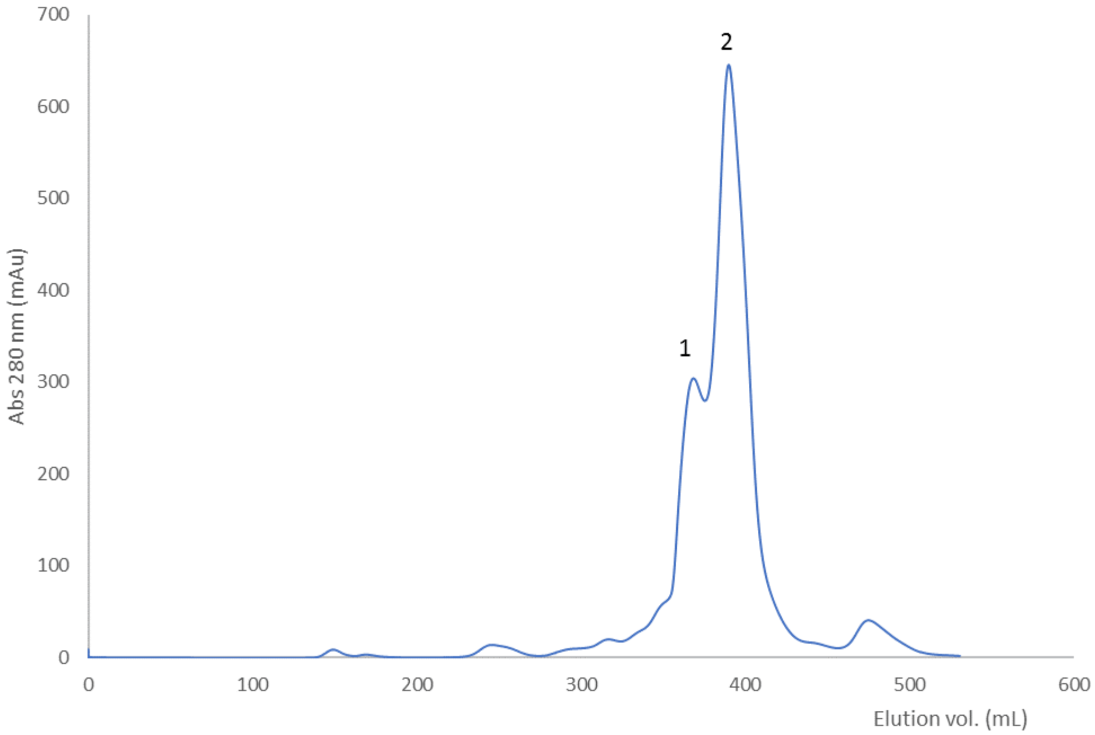

Fig. S1. Gel filtration chromatography of whole venom.
100 mg of *N. nigricollis* (Tanzania) venom was dissolved in 5 mL PBS and centrifuged prior to separation. This was loaded onto a 480 mL column of Superdex 200HR equilibrated in PBS. The column was operated at a flow rate of 2.0 mL/min and elution was monitored at 280 nm. The overlapping peaks for PLA_2 and 3FTX are numbered 1 and 2 respectively. These were pooled and then subjected to cation exchange chromatography (see Fig. S2).

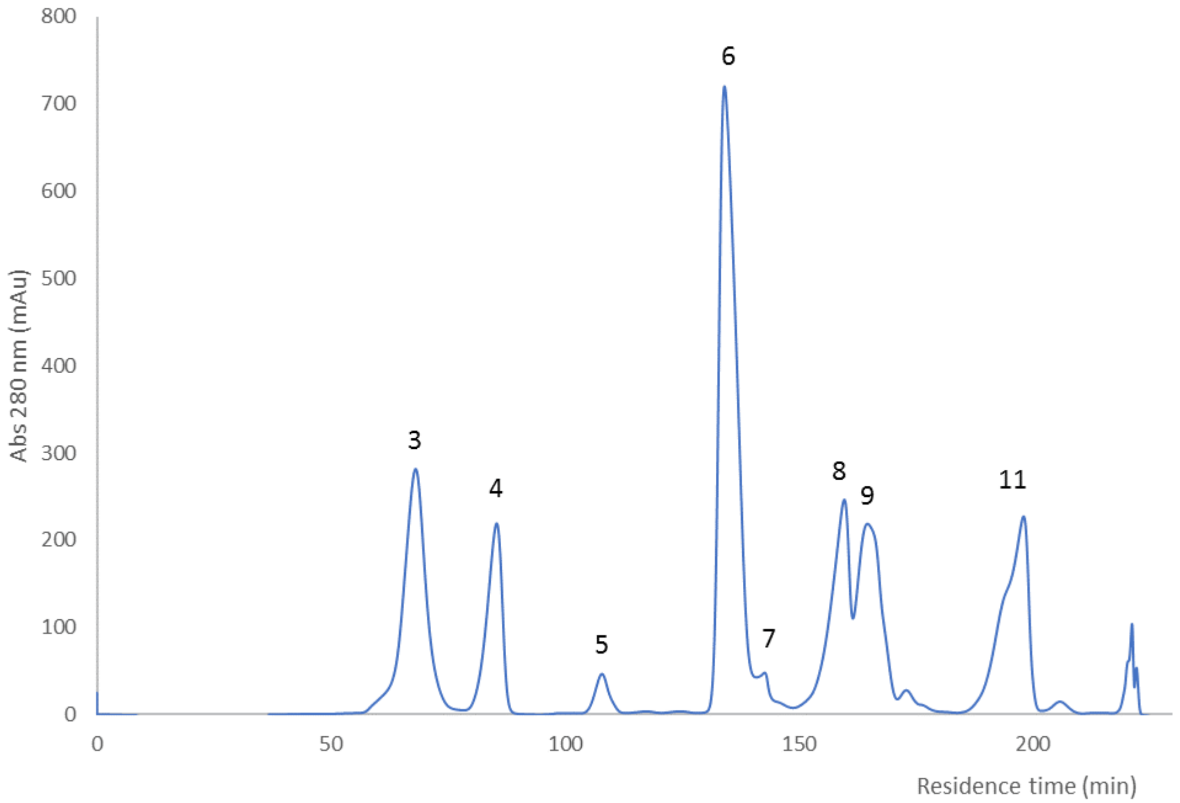

Fig. S2. Cation exchange chromatography of the PLA_2 and 3FTX fraction from GFC.
Peaks 1 and 2 from GFC were pooled and dialysed against 50 mM sodium phosphate, pH 6.0 and then applied to a 20 mL HPSP column equilibrated in the same buffer. Elution was carried using a 15 CV gradient of 0 – 0.7 M NaCl in 50 mM sodium phosphate, pH 6.0. Flow rate was 1.5 mL/min and elution was monitored at 280 nm. Peak 4 contained pure acidic PLA_2 (named acidic PLA_2 2). The major peaks, labelled 3, 6, 8, 9, and 11 were subject to further chromatographic steps.

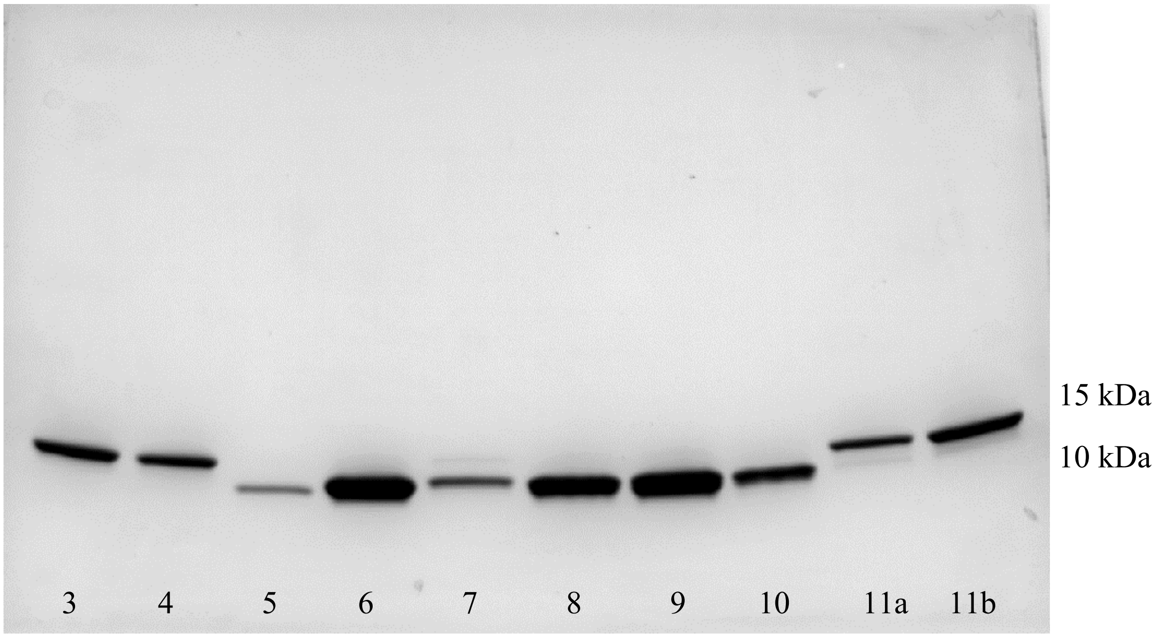

Fig. S3. SDS-PAGE analysis of peaks from cation exchange chromatography.

Seven μL samples were loaded onto 4-20% acrylamide gels (BioRad) and run using the Tris-glycine buffer system under reducing conditions. The gels were subsequently stained with Coomassie Blue R250. The position of relevant molecular weight markers is shown on the right (kDa). Chromatography peak numbers are indicated at the base of the figure. Peak 11 was run as two fractions, a and b, reflecting the wide asymmetric peak shape. Peak 10 contained overlapping material from peak 9, hence the band intensity is greater than would be expected for the height of peak 10.

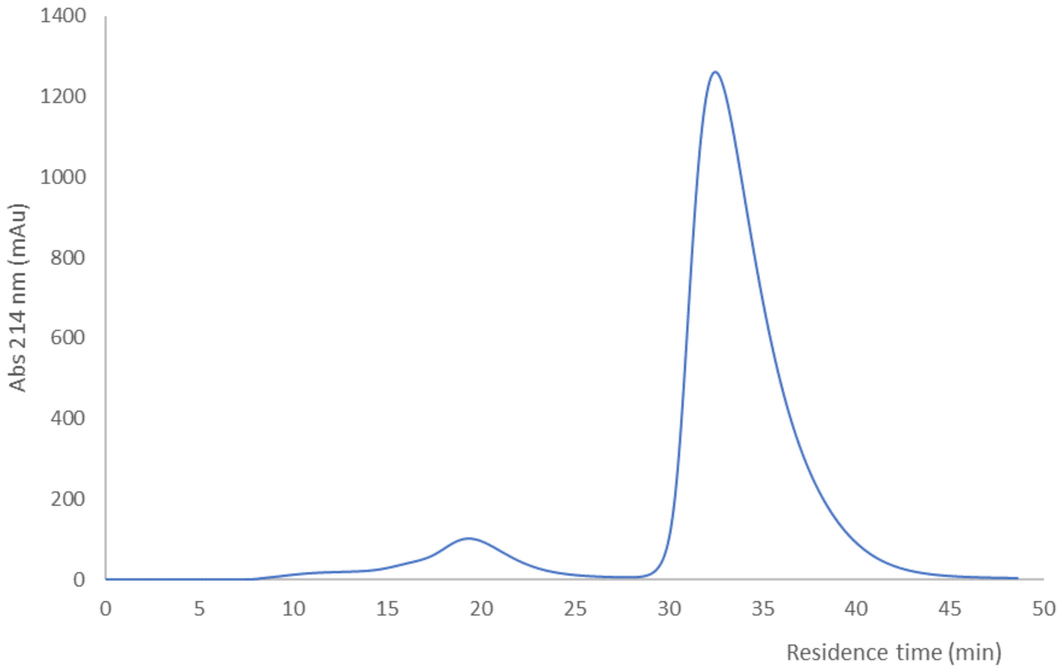

Fig. S4. Hydrophobic interaction chromatography of acidic PLA_2 (peak 3).
Peak 3 from the cation exchange chromatography step was loaded directly onto a 4.7 mL column of Phenyl Sepharose LS FF equilibrated in 25 mM sodium phosphate pH 7.2. Elution was carried out with a 0-100% (4CV) gradient of 25 mM sodium phosphate pH 7.2 to 25% ethanol in 25 mM sodium phosphate pH 7.2. The column was operated at 1.0 mL/min and elution was monitored at 214 nm.

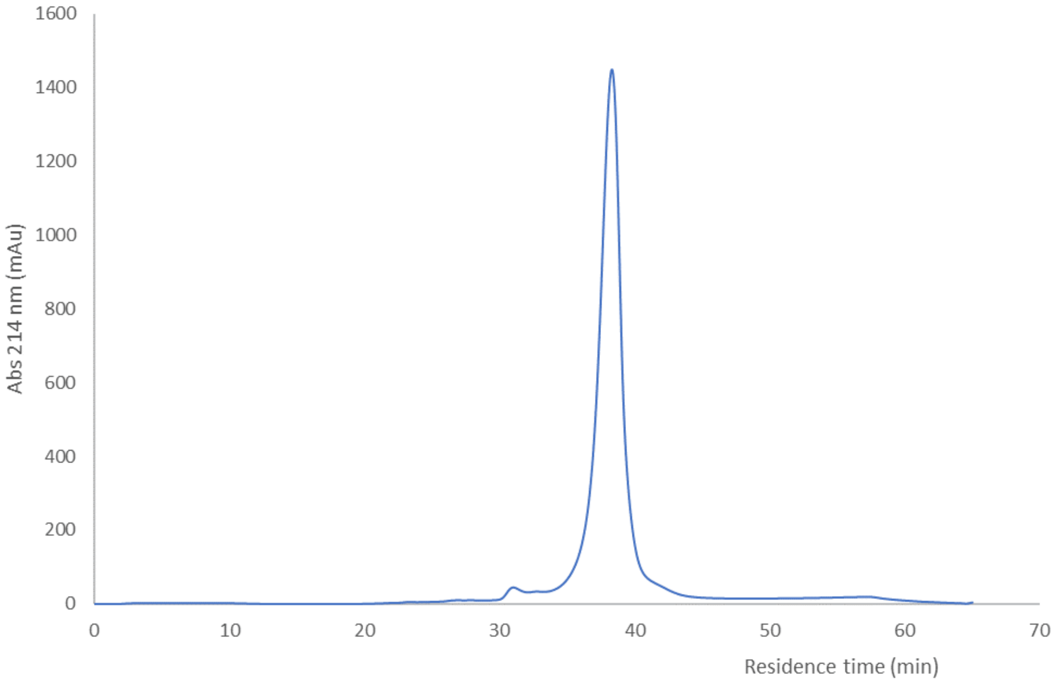

Fig. S5. Hydroxyapatite chromatography of basic PLA_2 (peak 11).

Peak 11 from the cation exchange chromatography step was dialysed against 5 mM sodium phosphate pH 6.8 and then loaded onto a 1 mL column of ceramic hydroxyapatite (CHT I) equilibrated in the same buffer. Using a flow rate of 0.5 mL/min, elution was carried out with gradient of 5 mM sodium phosphate pH 6.8 to 500 mM sodium phosphate pH 6.8, 0.15 M NaCl over 20 CV. Elution was monitored at 214 nm.

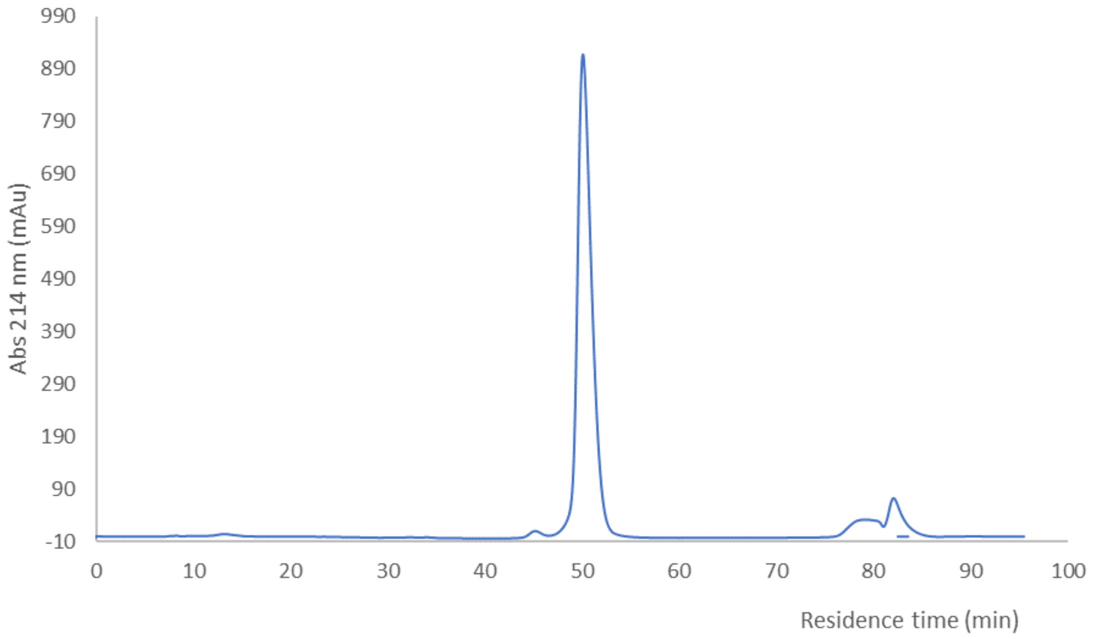

Fig. S6. Hydrophobic interaction chromatography of peak 6 3FTx cytotoxin 1.
The material in peak 6 from cation exchange chromatography was made to 1.5 M in NaCl and loaded onto a 10 mL Phenyl Superose column. Proteins were then eluted in a 5 CV gradient of 1.5 M NaCl in 25 mM sodium phosphate pH 7.2 to 30% (v/v) ethylene glycol in 25 mM sodium phosphate pH 7.2. The flow rate was 1 mL/min and elution was monitored at 214 nm.

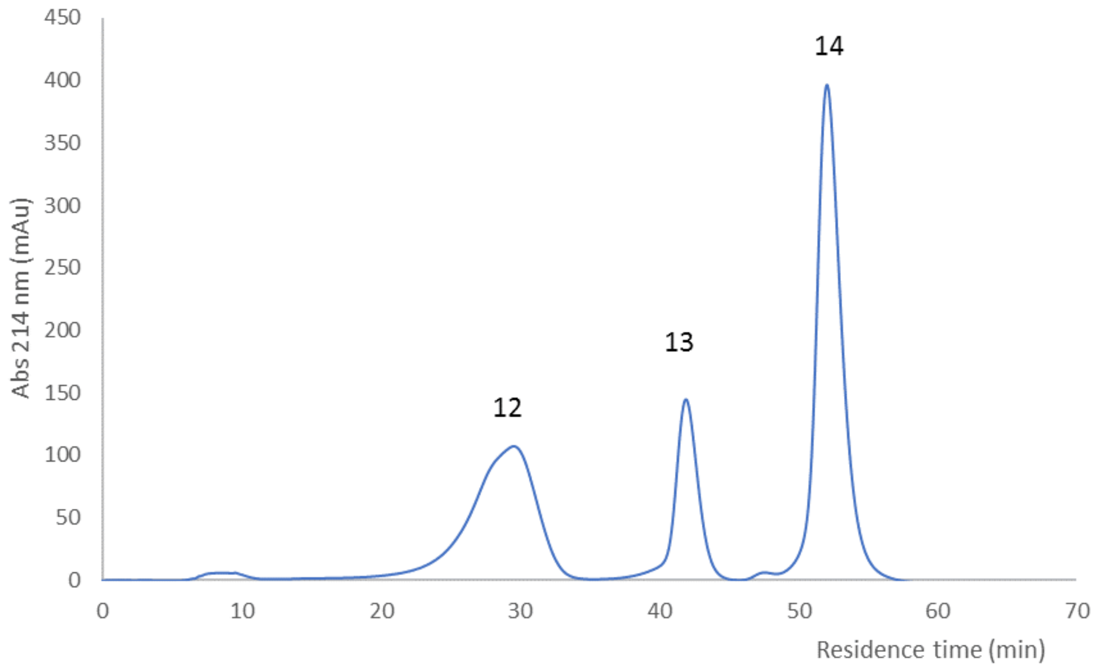

Fig. S7. Hydrophobic interaction chromatography of 3FTx cytotoxins 1v, 3 and 4.
The material in peak 8 and 9 from cation exchange chromatography was made to 1.2 M in NaCl and loaded onto a 10 mL Phenyl Superose column. Proteins were then eluted in a 5 CV gradient of 1.2 M NaCl in 25 mM sodium phosphate pH 7.2 to 30% (v/v) ethylene glycol in 25 mM sodium phosphate pH 7.2. The flow rate was 1 mL/min and elution was monitored at 214 nm. Peak 12, 13 and 14 respectively contained cytotoxin 4, cytotoxin 1v and cytotoxin 3.

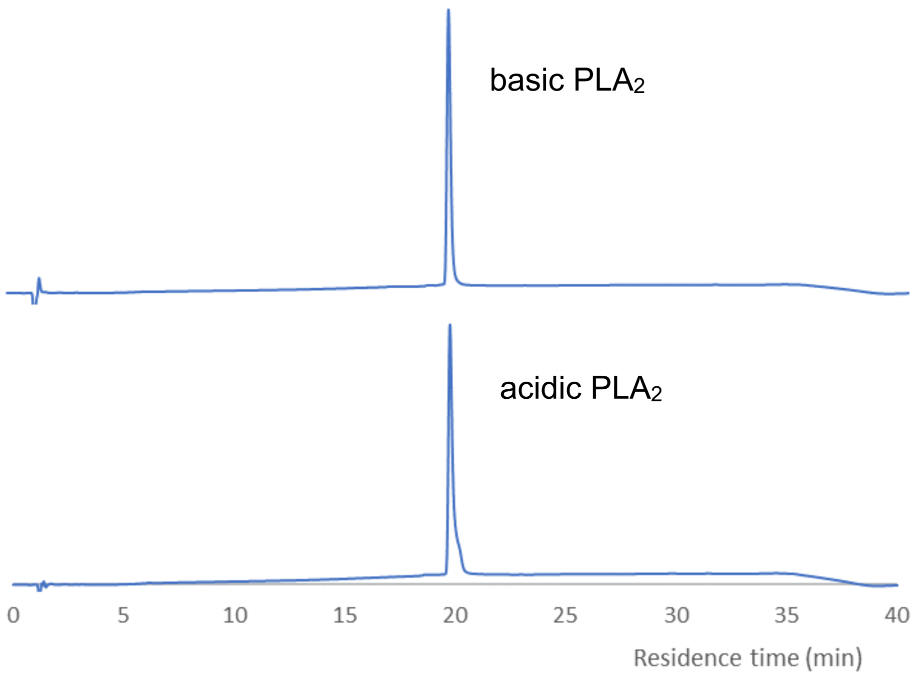

Fig. S8. Analytical RP-HPLC of purified PLA_2s.

RP-HPLC was performed on a Biobasic C4 column (2.1 x 150 mm). The flow rate was 0.2 mL/min and proteins were separated in a gradient of 0-65% acetonitrile in 0.1% trifluoroacetic acid, with monitoring at 214 nm (Y-axis, mAu units not shown). The acidic PLA_2 shown is acidic PLA_2 1; acidic PLA_2 2 had an identical profile on RP-HPLC and the two were combined to give the acidic PLA_2 pool used throughout this study.

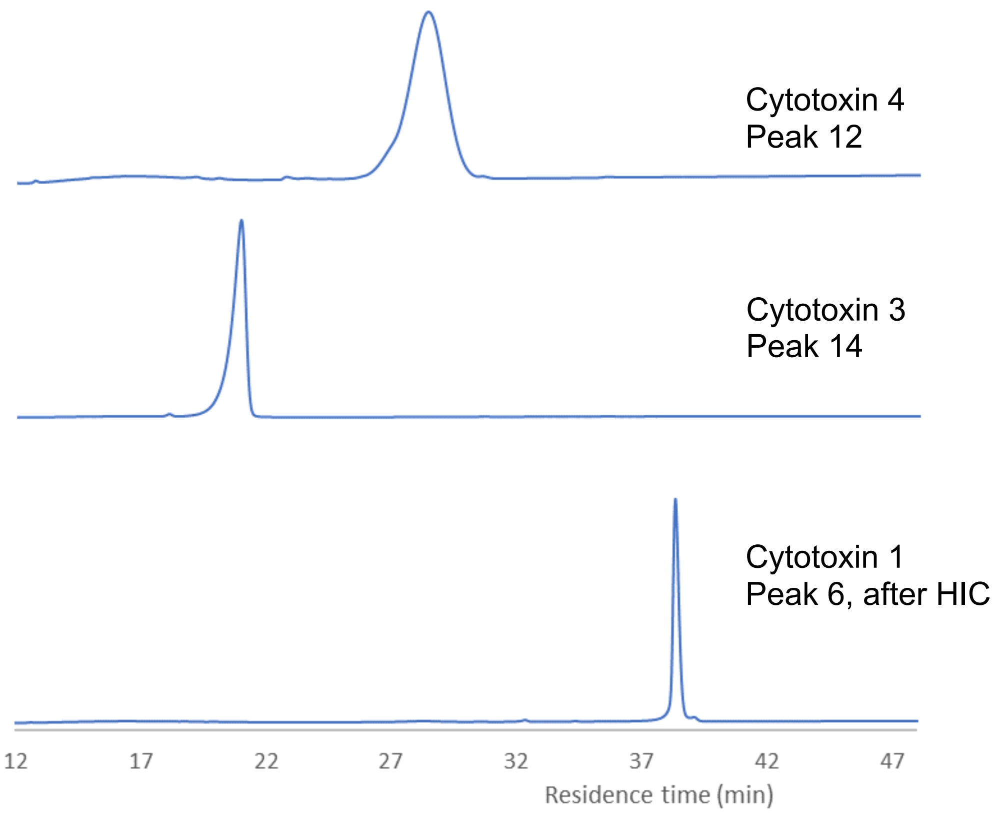

Fig. S9. Analytical RP-HPLC of purified 3FTx cytotoxins 1, 3 and 4.
RP-HPLC was performed on a Biobasic C4 column (2.1 x 150 mm). The flow rate was 0.2 mL/min and proteins were separated in a gradient of acetonitrile (0-25%/5 min, 25-50%/30 min, 50-90%/5 min) in 0.1% trifluoroacetic acid, with monitoring at 214 nm (Y-axis, mAu units not shown).

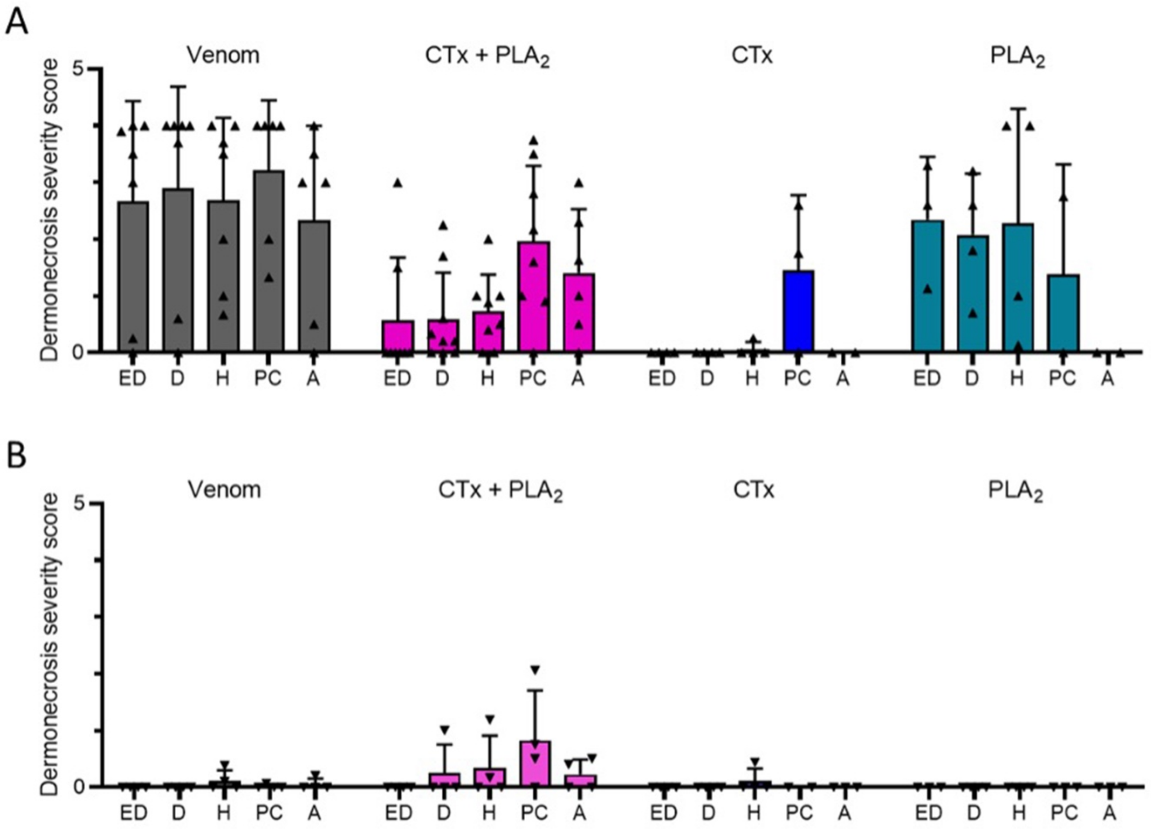

Fig. S10. Histopathological analysis of dermonecrotic skin lesions shows that microscopic pathology caused by *N. nigricollis* venom is prevented by the PLA_2 inhibitor varespladib. **A**) Histopathology analysis resulting in dermonecrosis severity scores for multiple skin layers are shown following intradermal dosing of mice with crude East African (Tanzania) *N. nigricollis* venom, the CTX + PLA_2 combination, and CTXs and PLA_2s separately. Key: ED, epidermis; D, dermis; H, hypodermis; PC, panniculus carnosus; A, adventitia. **B**) Co-injection of mice with the PLA_2 inhibitor varespladib significantly reduces damage scores across multiple skin layers. Data shown represents the mean damage score for each skin layer and error bars represent the corresponding standard deviations.

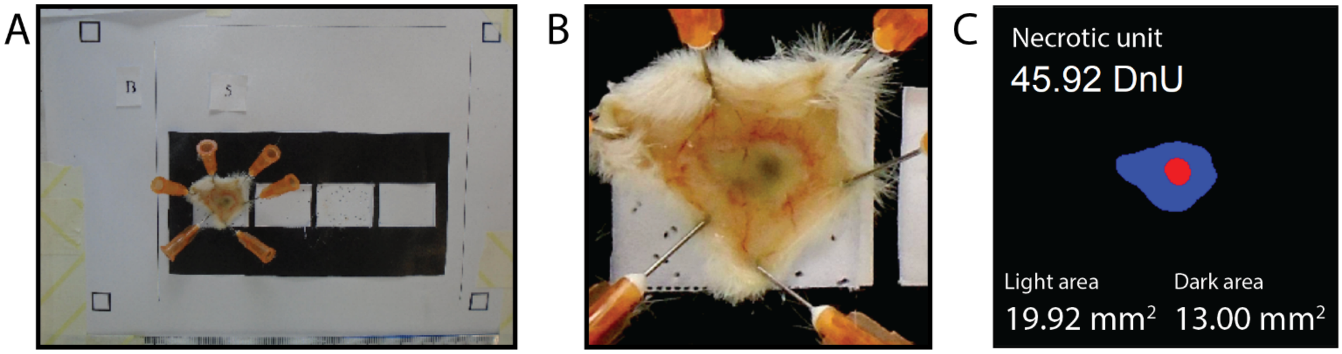

Fig. S11. Overview of the VIDAL workflow. First, **A**) the raw image is imported and automatically standardised. Thereafter, **B**) the lesion is identified and cropped out for further analysis. Finally, **C** the processed image is segmented and light and dark lesions automatically identified. This information is used to calclulate dermonecrotic units (DnUs) for each identified lesion, with a 2:1 weighting of dark to light lesion areas.

Table S1. Summary of MS analysis and identification of the purified toxins.

Chromatography peak no.		Intact mass	MS/MS ID					
CatX	HIC	(Mono, Da)	Protein ID	Entry Name	Length	Percent Coverage	Organism	Protein Description
3		13,172	P00602	PA2A1_NAJMO	118	85.6	Naja mossambica OX=8644	Acidic phospholipase A2 CM-I
4		13,287	P00602	PA2A1_NAJMO	118	85.6	Naja mossambica OX=8644	Acidic phospholipase A2 CM-I
11		13,249	P00605	PA2B4_NAJNG	118	93.2	Naja nigricollis OX=8654	Phospholipase A2 'basic'
6		6,818	P01468	3SA1_NAJPA	60	100	Naja pallida OX=8658	Cytotoxin 1
8+9	12	6,707	P01452	3SA4_NAJMO	60	63.3	Naja mossambica OX=8644	Cytotoxin 4
8+9	13	6,817	P01468	3SA1_NAJPA	60	100	Naja pallida OX=8658	Cytotoxin 1
8+9	14	6,884	P0DSN1	3SAN_NAJNG	60	96.7	Naja nigricollis OX=8654	Naniproin [cytotoxin 3]

Table S2. Dermonecrotic lesions from mice dosed with *N. nigricollis* venom and toxins, with and without varespladib.

Mice were intradermally injected with East African (Tanzania) *N. nigricollis* venom (63 µg), or proportional amounts of purified CTx + PLA_2s (37.8 + 16.4 µg), purified CTx-only (37.8 µg), and purified PLA_2s-only (16.4 µg) either alone, or pre-incubated with the PLA_2-inhibitor varespladib (19 µg). Mice were culled at 72 h post-injection, lesions excised, and photographs taken. White scale bars represent 5 mm.

**Venom**	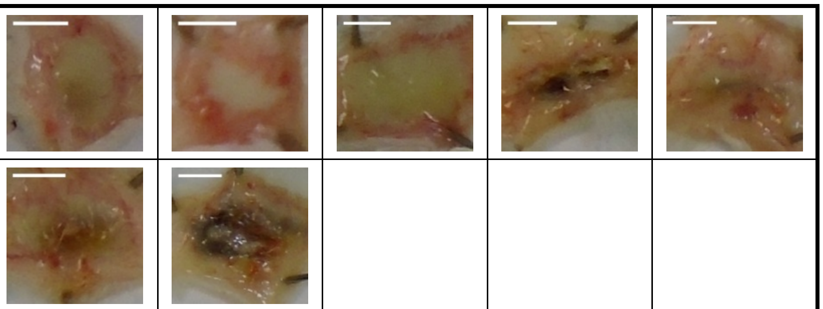
**Venom + varespladib**	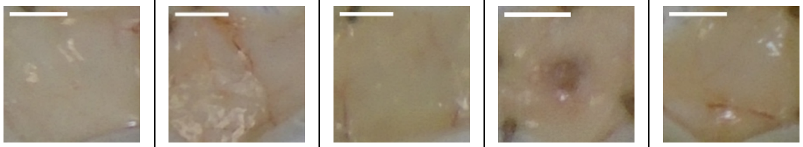
**CTXs + PLA_2s**	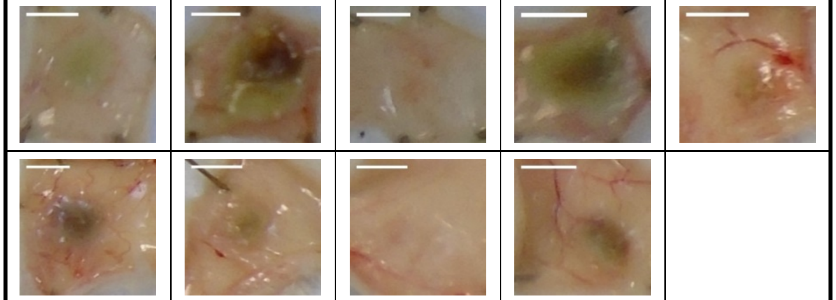
**CTXs + PLA_2s + varespladib**	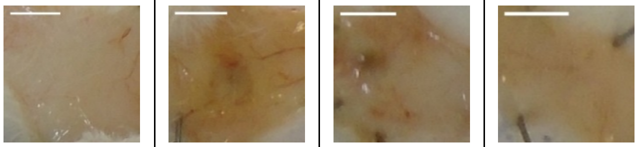
**CTXs-only**	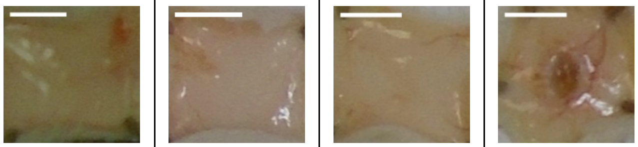
**CTXs-only + varespladib**	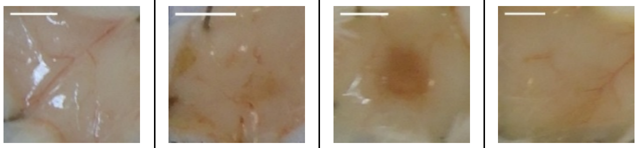
**PLA_2s-only**	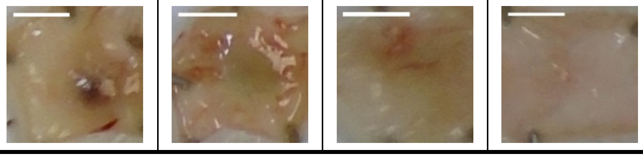

**PLA_2s + varespladib**	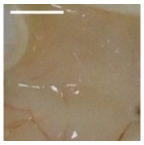	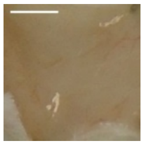	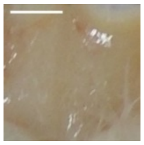	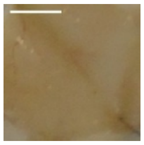	

Table S3. Dermonecrotic lesions from mice dosed with *N. pallida* venom, with and without varespladib.

Mice were intradermally injected with *N. pallida* venom (25 µg) either alone, or pre-incubated with the PLA_2-inhibitor varespladib (19 µg). Mice were culled at 72 h post-injection, lesions excised, and photographs taken. White scale bars represent 5 mm.

**Venom**	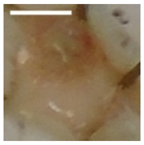	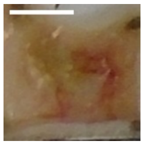	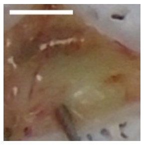	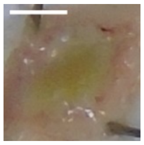	
**Venom + varespladib**	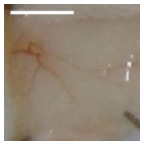	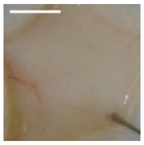	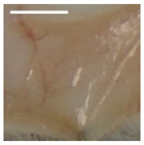	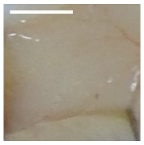	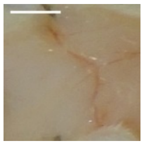

Data S1-7. All underpinning data used for figures presented in the manuscript and SI file. Data for each figure is contained within an individually labelled file, with data for individual panels labelled within each spreadsheet. The data files track to the figures as follows:

Bartlett Data S1 → Figure 1
Bartlett Data S2 → Figure 2
Bartlett Data S3 → Figure 3
Bartlett Data S4 → Figure 4
Bartlett Data S5 → Figure 5
Bartlett Data S6 → Figure 6
Bartlett Data S7 → Figure S10
